# Multipotent adult progenitor cells induce regulatory T cells and promote their suppressive phenotype via TGFβ and monocyte-dependent mechanisms

**DOI:** 10.1038/s41598-021-93025-x

**Published:** 2021-06-30

**Authors:** Alice Valentin-Torres, Cora Day, Jennifer M. Taggart, Nicholas Williams, Samantha R. Stubblefield, Valerie D. Roobrouck, Jelle Beyens, Anthony E. Ting

**Affiliations:** 1 Athersys, Inc. Cleveland, 3201 Carnegie Ave., Cleveland, OH 44115 USA; 2 ReGenesys BV, Leuven, Belgium

**Keywords:** Regulatory T cells, Adult stem cells, Monocytes and macrophages

## Abstract

Dysregulation of the immune system can initiate chronic inflammatory responses that exacerbate disease pathology. Multipotent adult progenitor cells (MAPC cells), an adult adherent bone-marrow derived stromal cell, have been observed to promote the resolution of uncontrolled inflammatory responses in a variety of clinical conditions including acute ischemic stroke, acute myocardial infarction (AMI), graft vs host disease (GvHD), and acute respiratory distress syndrome (ARDS). One of the proposed mechanisms by which MAPC cells modulate immune responses is via the induction of regulatory T cells (Tregs), however, the mechanism(s) involved remains to be fully elucidated. Herein, we demonstrate that, in an in vitro setting, MAPC cells increase Treg frequencies by promoting Treg proliferation and CD4^+^ T cell differentiation into Tregs. Moreover, MAPC cell-induced Tregs (miTregs) have a more suppressive phenotype characterized by increased expression of CTLA-4, HLA-DR, and PD-L1 and T cell suppression capacity. MAPC cells also promoted Treg activation by inducing CD45RA^+^ CD45RO^+^ transitional Tregs. Additionally, we identify transforming growth factor beta (TGFβ) as an essential factor for Treg induction secreted by MAPC cells. Furthermore, inhibition of indoleamine 2, 3-dioxygenase (IDO) resulted in decreased Treg induction by MAPC cells demonstrating IDO involvement. Our studies also show that CD14^+^ monocytes play a critical role in Treg induction by MAPC cells. Our study describes MAPC cell dependent Treg phenotypic changes and provides evidence of potential mechanisms by which MAPC cells promote Treg differentiation.

## Introduction

Tregs are indispensable players of immune regulation by maintaining self-tolerance and homeostasis. Tregs are characterized as natural Tregs (nTregs), which are developed in the thymus during embryonic state, or induced Tregs (iTregs) that arise from effector T cells in the periphery, preferentially during inflammatory conditions^1^. Tregs express both CD4 and CD25 surface antigens as well as the transcription factor, FoxP3, a critical gene involved in Treg development and function^2,3^. Treg deficient mice suffer fatal autoimmunity called “scurfy mice”^4^. Similarly, humans born with dysfunctional FoxP3 develop an autoimmune syndrome called immunodysregulation polyendocrinopathy enteropathy X-linked (IPEX), which is characterized by severe enteropathy, endocrinopathy, and eczematous dermatitis^5,6^. Tregs control inflammation and modulate the immune system by several mechanisms which can be categorized as: (1) secretion of anti-inflammatory factors such as interleukin 10 (IL-10), interleukin 35 (IL-35), and TGFβ; (2) metabolic disruption by cyclic adenosine monophosphate (cAMP), CD39, and CD73; (3) inhibition of antigen presenting cell maturation; and (4) induction of effector T cell death by interleukin 2 (IL-2) consumption and granzyme and perforin cytolysis^7,8^. Furthermore, Tregs express co-inhibitory receptors, including cytotoxic T lymphocyte associated protein 4 (CTLA-4) and program cell death protein 1 ligand (PD-L1), that further support Treg immune regulatory properties. Due to its ability to reduce inflammation and modulate the immune system, the use of Tregs as therapy to treat autoimmune diseases is currently being explored^9,10^.

MAPC cells are adult bone marrow derived adherent cells with immunomodulatory properties that reduce inflammation by regulation of immune system functions^11^. MAPC cells inhibit allogeneic T-cells in a mixed lymphocyte reaction (MLR) and suppress an allogeneic reaction between two mismatched lymphocyte populations^12,13^. MAPC cells inhibit allogeneic cell and memory response mediated T-cell proliferation in vitro in a dose-dependent manner^14^. In vitro and in vivo studies have also demonstrated that MAPC cells suppress T cell homeostatic expansion driven by IL-7 via prostaglandin E2 (PGE-2)^15,16^. In rat models of traumatic brain injury or stroke, MAPC cell treatment significantly increases Treg frequencies in the spleen and blood^17–19^. Concurrently, MAPC cell treatment reduces proliferation of both CD4^+^ and CD8^+^ T effector cells^17^. MAPC cell administration also leads to a reduction of pro-inflammatory cytokines in a sheep model of hypoxic ischemia and rat models of stroke or traumatic brain injury^17–20^.

MAPC cells share many immunomodulatory functions with mesenchymal stem cells (MSCs), however, they are phenotypically and functionally distinct cell types^21^. MAPC cells can be differentiated from MSCs by size and morphological features, differential expression of surface markers such as CD140a and CD140b, and their production of CXCL5^21–23^. Additionally, MAPC cells are cultured in hypoxic conditions and can be expanded to higher population doublings than MSCs making large scale manufacturing more feasible. In a clinical study evaluating the administration of MultiStem^®^, a clinical grade product of MAPC cells, in patients receiving a liver transplant, a transient upregulation of Tregs in the blood was observed^24^. MultiStem is currently under clinical evaluation to treat acute ischemic stroke (NCT03545607), ARDS (NCT02611609), and trauma (NCT04533464). In the case of ischemic stroke, a Phase 2 clinical trial revealed that MultiStem treatment not only improved clinical outcomes but also reduced inflammation characterized by decreasing T effector cells and pro-inflammatory cytokines levels in the blood^25^.

While MAPC cell-dependent induction of Tregs has been observed^17,18,24,26^, the mechanism(s) by which MAPC cells increase Tregs remain to be fully elucidated. In this study, we investigated MAPC cell induction of Tregs in vitro and examined the effects of MAPC cells on Treg proliferation, characterization of their suppressive phenotype, and analysis of their expression of CD45 isoforms. We also evaluated the involvement of factors secreted by MAPC cells in the induction of Tregs. These results provide greater insight into the mechanistic pathways in which MAPC cells may modulate inflammation and immune responses in the setting of acute inflammatory diseases including ischemic stroke, ARDS, GvHD and AMI.

## Results

### Co-culture of PBMCs with MAPC cells increase Tregs

**Figure 1:**
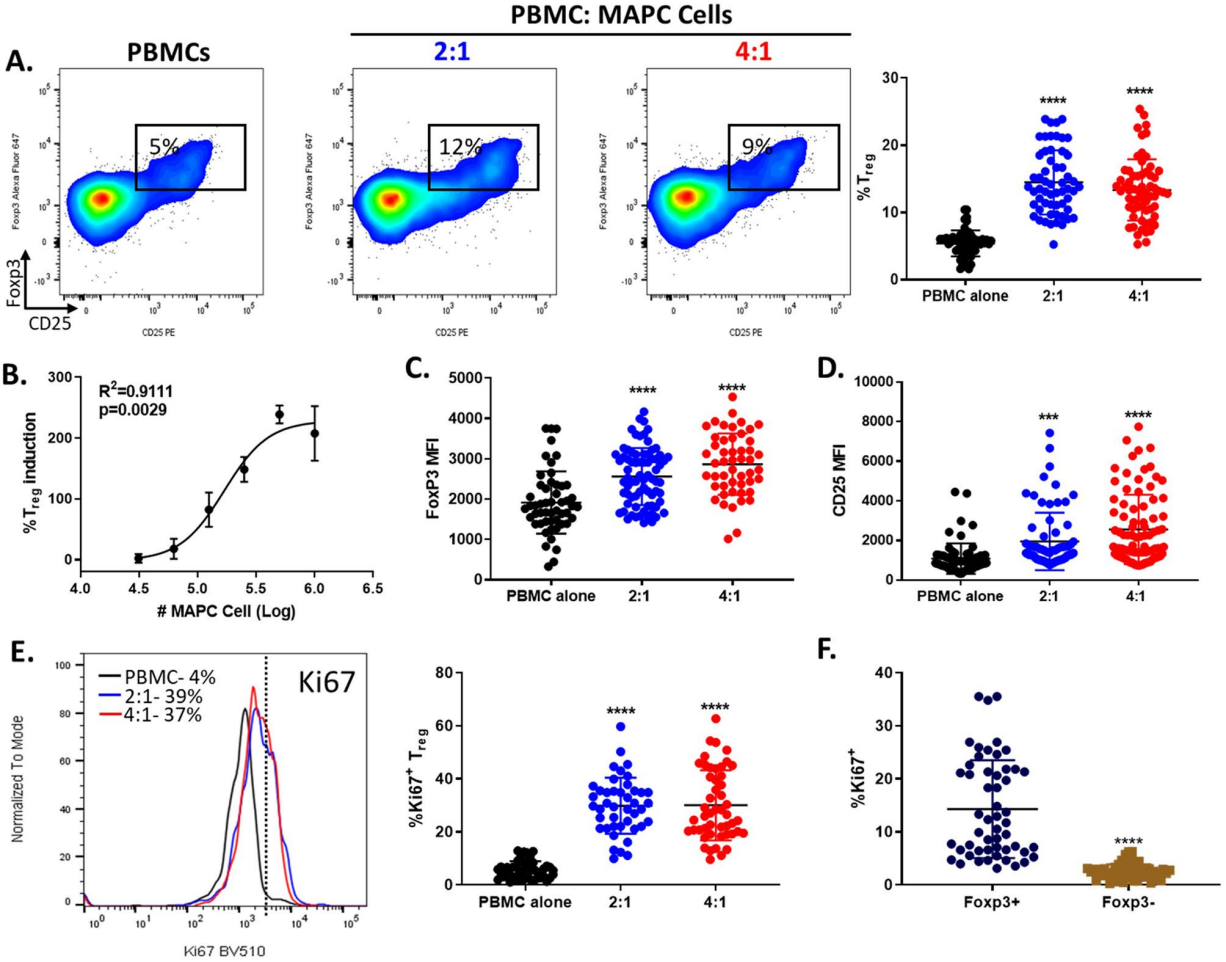
Co-culturing PBMCs and MAPC cells increase Treg frequency. (**A**) Representative dot plots depicting the percentages of Tregs (CD3^+^ CD4^+^ FoxP3^+^ CD25^+^) in PBMC alone and PBMCs co-cultured with MAPC cells for 7 days (2:1 and 4:1 PBMC:MAPC cell ratios). Graph shows quantification of MAPC cell dependent Treg induction. Each symbol represents one n from multiple independent experiments. (**B**) Dose dependent curve of Treg induction. Quantification of (**C**) FoxP3 and (**D**) CD25 MFI on Tregs. (**E**) Representative histogram depicting Ki67 expression on Tregs from PBMC (black line), 2:1 (blue line), and 4:1 (red line) co-cultures. FMO shown as dotted line. Numbers represent the percent Ki67 positive within the Treg population. Graph shows quantification of Ki67 expression on Tregs. Data represent mean ± SD from pooled samples of 16 independent experiments with 10 PBMC different donors and 3 different MAPC cell donors. P-values were determined by One-way ANOVA with a Tukey’s multiple comparisons test reference to the control PBMC alone (****p < 0.0001 and ***p < 0.001). (**F**) Ki67 expression on FoxP3 positive (navy) and FoxP3 negative (gold) population from 2:1 PBMC:MAPC cell co-cultures. P-value was determined by unpaired *t* test.

In vivo and in vitro, MAPC cells have been shown to increase regulatory T cells (Tregs)^17,18,24,26^. However, the mechanisms by which MAPC cells augment Tregs remains to be fully understood. To understand and characterize MAPC cell induced Tregs (miTreg) in vitro, peripheral blood mononuclear cells (PBMCs) were co-cultured with allogeneic MAPC cells at different PBMC:MAPC cell ratios. Since T cell activation can result in transient expression of FoxP3 and CD25 on effector T cells, making the identification of Tregs more challenging, our studies were performed in the absence of T cell stimulation^27–29^. The percentage of Tregs (CD3^+^ CD4^+^ CD25^+^ FoxP3^+^, see Supplemental Fig. 1 for gating strategy) was determined by flow cytometry at day 7 (Fig. 1). As seen in vivo, allogeneic MAPC cells increased the percentage of Tregs by approximately 2.7-fold at 2:1 and 2.5-fold at 4:1 PBMC:MAPC cell ratios, respectively (Fig. 1A). MAPC cell induction of Tregs was also assessed at day 4, 5, and 14. At day 4, co-culture of PBMCS with MAPC cells at 2:1 PBMC:MAPC cell ratio increased Tregs frequencies approximately 80% while no Treg induction was observed at 4:1 (Supplemental Fig. 2A). At day 5, MAPC cells also increased Treg frequencies in both 2:1 and 4:1 PBMC:MAPC cell ratios, however, maximum Treg induction was observed at day 7. Augmented cell death was observed after 14-day co-culture (data not shown). Given that in vivo studies have demonstrated that MAPC cells are undetected approximately 7 days after administration^20,30,31^ and maximal MAPC cell mediated Treg induction was observed at day 7, induction of Tregs by MAPC cells was studied at day 7. Increased Treg counts were also observed after co-culture with MAPC cells at both day 5 and day 7 (Supplemental Fig. 2B). Further dilutions of MAPC cell concentration demonstrated that MAPC cell induction of Tregs is dose dependent (Fig. 1B). Increased FoxP3 and CD25 expression on Tregs have been shown to correlate with increased anti-inflammatory capacity^32–34^. Thus, the FoxP3 and CD25 median fluorescent intensity (MFI) within the Treg population was examined (Fig. 1C,D). Interestingly, miTregs displayed increased FoxP3 and CD25 expression than Tregs from PBMCs cultured alone. Comparable to Tregs, miTregs were Helios positive (~ 80%) and CD127^low^, further confirming that these cells express other classical Treg lineage markers along with FoxP3 and CD25 (Supplementary Fig. 2D,E).

To study whether MAPC cells can drive the differentiation of CD4^+^ T cells into Tregs, CD25^+^ cells were depleted using magnetic bead separation and CD25 negative PBMCs were co-cultured with MAPC cells. We found that MAPC cells increased the percent of Tregs by 2.6-fold, suggesting that MAPC cells can induce the conversion of CD4^+^ T cells into Tregs (Supplemental Fig. 3B). Collectively, these data demonstrate that MAPC cell interactions with PBMCs result in the induction of Tregs.

### MAPC cells augment Tregs proliferation in vitro

To examine if MAPC cells induce Treg expansion_,_ Treg proliferation was determined by assessing the expression of Ki67 as a surrogate marker of proliferation in the presence or absence of MAPC cells (Fig. 1E). Indeed, miTregs showed elevated Ki67 expression than Tregs from PBMCs cultured alone. Increased Ki67 expression was only observed at day 7 post co-culture (Supplemental Fig. 2C). To confirmed that MAPC cells indeed induce Treg proliferation, PBMCs were labeled with a cell proliferation dye (cell proliferation dye efluor 450) and after 7 days in culture, Treg proliferation was assessed (Supplementary Fig. 2F). Dilution of the cell proliferation dye was only observed on Tregs from PBMCs co-cultured with MAPC cells, confirming that MAPC cells indeed induce Treg proliferation. MAPC cells also increased Ki67 expression on Tregs when co-cultured with CD25 negative PBMCs (Supplemental Fig. 3C). Ki67 induction by MAPC cells was restricted to the FoxP3^+^ Tregs and not FoxP3^−^ CD4^+^ T cells (Fig. 1F). Together, these data suggest that MAPC cells preferentially promote the expansion of Tregs but not FoxP3^−^ CD4^+^ T cells, as previously reported^14^.

Analysis of PBMC:MAPC cell co-culture supernatant revealed that IL-2 levels were increased when compared to supernatants from PBMCs cultured alone (Supplemental Fig. 2G). Low IL-2 levels were detected in supernatants from PBMCs and MAPC cells cultured alone. To identify the source of IL-2, cell cultures were treated with Golgistop for 18 h and IL-2 was measured intracellularly by flow cytometry. IL-2 was primarily secreted by Tregs (Supplemental Fig. 2H). Conversely, IL-2 was not detected on FoxP3^−^ CD4^+^ T cells, MAPC cells, or monocytes (data not shown). These data suggest that MAPC cells induce Treg activation, thereby promoting their secretion of IL-2 which can potentially contribute to Treg proliferation.

### Increased suppressive phenotype and function in miTregs

**Figure 2:**
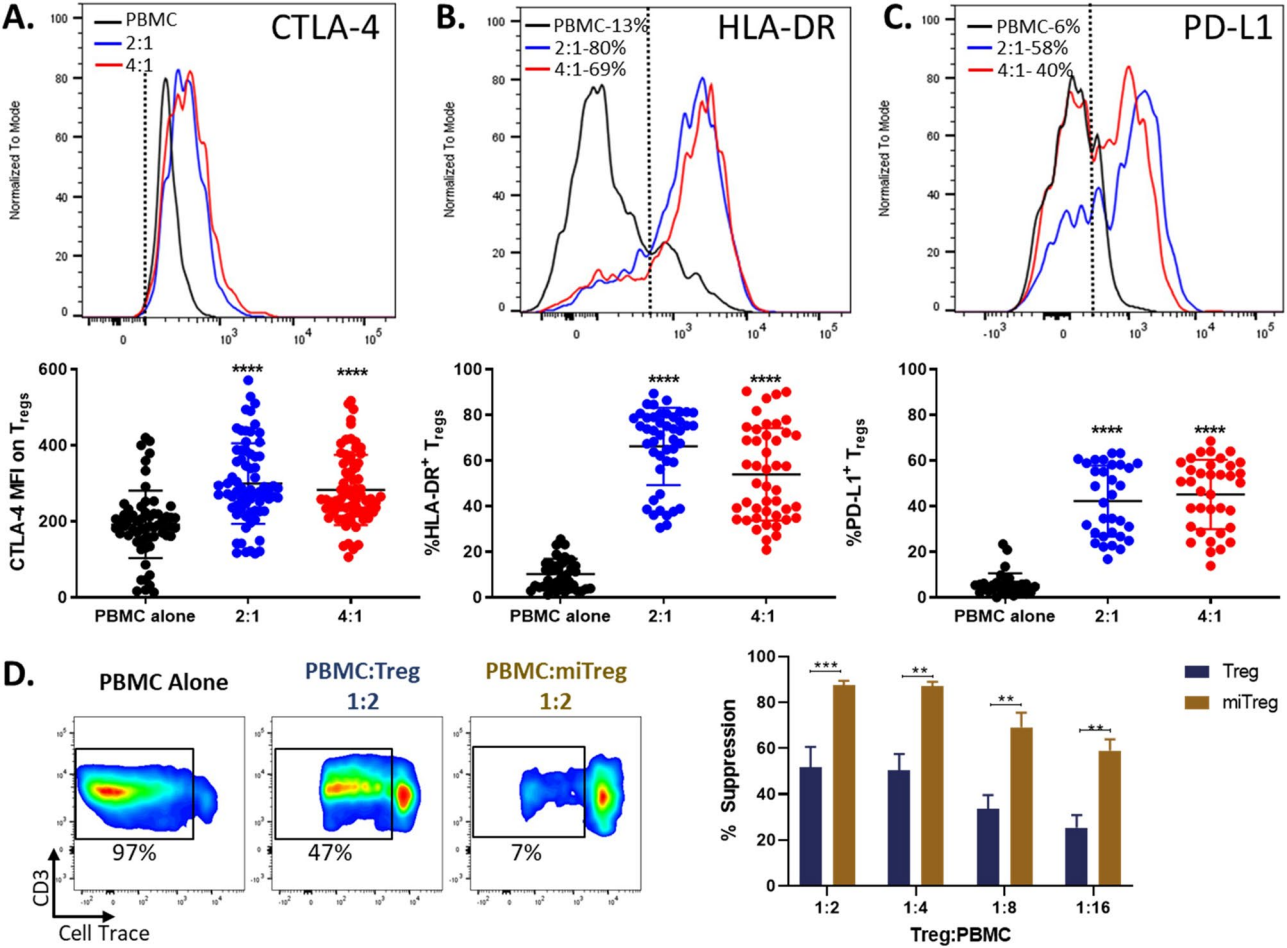
MAPC cells increase Treg suppressive phenotype and function capacity. Representative histogram depicting (**A**) CTLA-4, (**B**) HLA-DR, and (**C**) PD-L1 expression on Tregs from PBMC (black line), 2:1 (blue line), and 4:1 (red line) 7-day co-cultures. FMO shown as dotted line. Numbers represent the percent positive of corresponding marker within the Treg population. Graphs showing quantification of CTLA-4 (MFI), HLA-DR, and PD-L1 expression on Tregs. (**D**) Representative dot plots depicting CD3^+^ T cell proliferation in PBMCs cultured alone or PBMCs co-cultured with either Tregs or miTregs (1:2) after stimulation with anti-CD3/CD28. Quantification of suppression of T cell proliferation by Tregs (blue) or miTregs (gold) normalized to percent suppression. Data comprises 6 independent experiments from 6 healthy individual blood donors. Statistical analysis was performed using One-way ANOVA with a Tukey’s multiple comparisons test reference to the control PBMC alone (****p < 0.0001, ***p < 0.001, and **p < 0.01).

Increased expression of CTLA-4, HLA-DR, and PD-L1 on Tregs has been shown to correlate with a potent suppressive phenotype^35–38^. The expression of these markers on miTregs was assessed by flow cytometry and compared to Tregs from PBMCs (Fig. 2). miTregs had an increased expression of CTLA-4 (Fig. 2A), HLA-DR (Fig. 2B), and PD-L1 (Fig. 2C) compared to PBMC Tregs. MAPC cell induction of CTLA-4, HLA-DR, and PD-L1 expression was restricted to Tregs and not FoxP3^−^ CD4^+^ T cells (Supplemental Fig. 4). HLA-DR expression was also increased by MAPC cells on Tregs when co-cultured with CD25 negative PBMCs (Supplemental Fig. 2D). To investigate the effects of MAPC cells on Treg suppressive function, a T cell suppression assay was performed (Fig. 2D). miTregs suppressed T cell proliferation more efficiently than PBMC derived Tregs in all Treg: PBMC ratios tested, correlating with an increased suppressive phenotype. In addition to suppressing T cell proliferation more efficiently, miTregs also produced increased levels of TGFβ, also correlating with increased suppressive capacity (Supplemental Fig. 2I). Together, these data demonstrate that MAPC cells not only increase the frequency of Tregs and their expansion but also its activation and suppressive status.

### MAPC cells activate Tregs thereby increasing CD45RA^+^ CD45RO^+^ transitional population

**Figure 3:**
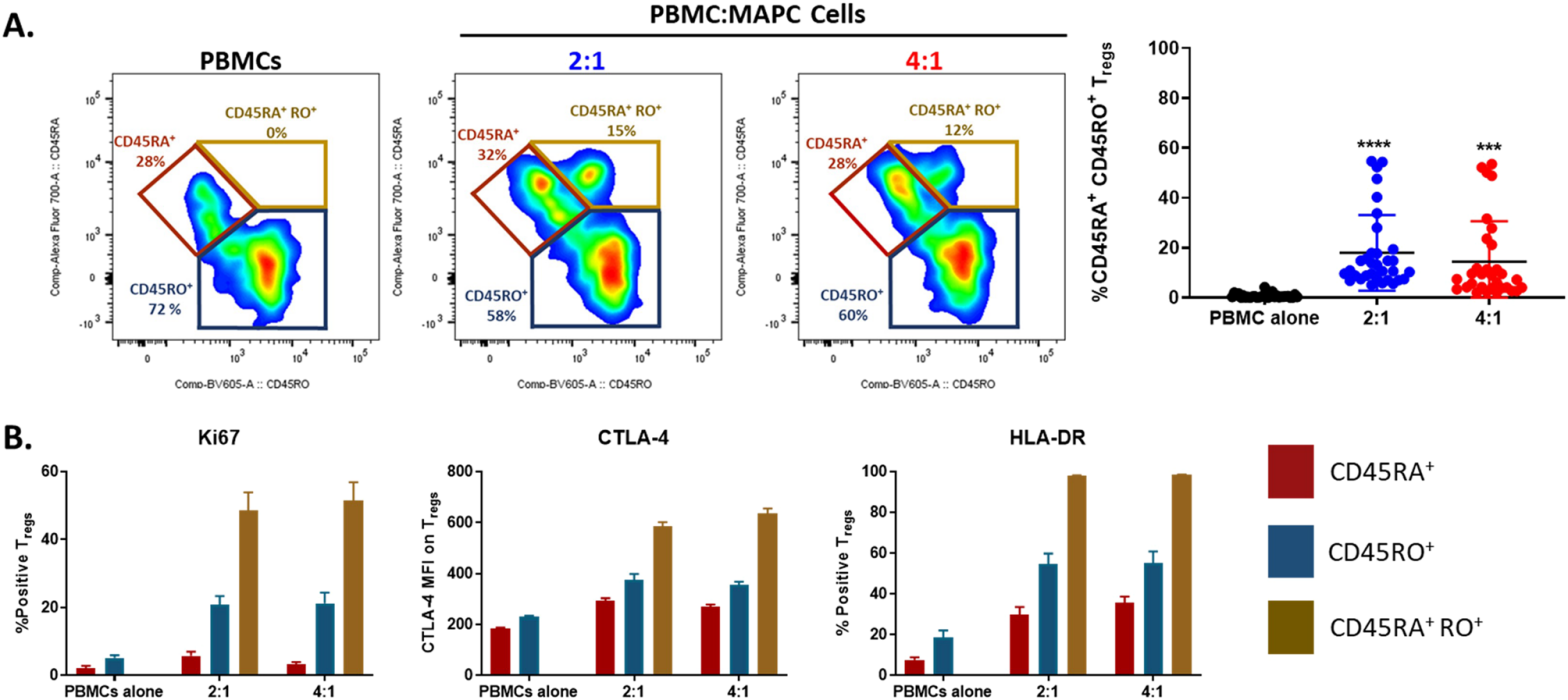
MAPC cells activate Tregs by increasing CD45RA^+^ CD45RO^+^ transitional cells. (**A**). Representative dot plots depicting CD45RA and CD45RO expression on Tregs from PBMC, 2:1, and 4:1 PBMC:MAPC cell 7-day co-cultures. Numbers represent the percent positive cells within the Treg population. Graph represents quantification of CD45RA^+^ CD45RO^+^ (transitional) frequencies within the Treg population. (**B**) Ki67, CTLA-4, and HLA-DR expression on CD45RA^+^ (crimson), CD45RO^+^ (blue), and CD45RA^+^ RO^+^ (gold) Tregs. Data represent mean ± SD from pooled samples of 5 independent experiments with 5 PBMC and 3 MAPC cell donors. Statistical analysis was performed using One-way ANOVA with a Tukey’s multiple comparisons test in reference to the control PBMC alone (****p < 0.0001 and ***p < 0.001).

Isoforms of CD45, CD45RA and CD45RO are surrogate markers of naïve and memory T cells respectively^39,40^. Upon activation, naïve CD45RA^+^ T cells upregulate CD45RO and HLA-DR and overtime, activated T cells lose their CD45RA expression, thus turning into an activated memory T cell (CD45RO^+^ HLA-DR^+^ CD45RA^−^)^41–43^. Approximately, 70–95% of Tregs are CD45RO^+^ and the frequency of these cells increases with age^41–43^. Furthermore, activation and proliferation of CD45RA^+^ Tregs causes loss of CD45RA expression and gain of CD45RO turning them into memory/activated Tregs^41,44^. To evaluate whether MAPC cells favorably induce activation and proliferation of naïve or memory Tregs, the expression of CD45RA and CD45RO on miTregs was determined (Fig. 3). As previously described, Tregs from PBMCs cultured alone preferentially expressed CD45RO; however, in the presence of MAPC cells, a portion of Tregs adopt a transitional phenotype (CD45RA^+^ CD45RO^+^) (Fig. 3A) suggesting that MAPC cells are activating CD45RA^+^ naïve Tregs. To characterize the activation and proliferation state of miTregs, the expression of Ki67, CTLA-4, and HLA-DR was assessed and compared among the CD45RA^+^ (naïve), CD45RO^+^ (memory), and CD45RA^+^ RO^+^ (transitional) Treg populations. MAPC cells increased the proliferation and activation of naïve, memory, and transitional Tregs. However, transitional Tregs (CD45RA^+^ CD45RO^+^) had elevated expression of Ki67, CTLA-4, and HLA-DR (Fig. 3B), correlating with the transitional activated phenotype described in effector T cells^39,40^. Together, these data suggest that MAPC cells activate and increase the proliferation of both naïve and memory Tregs, and activation of naïve Tregs leads to increased frequency of transitional CD45RA^+^ CD45RO^+^ Tregs.

To confirm that the CD45RA^+^ CD45RO^+^ transitional Tregs arise from MAPC cell activation of CD45RA^+^ Treg population, both CD45RA^+^ and CD45RO^+^ CD4^+^ T cells were enriched using microbead isolation and co-cultured with isolated CD14^+^ monocytes and MAPC cells for 7 days (Supplementary Fig. 5). Co-culture of MAPC cells with either CD45RA^+^ or CD45RO^+^ CD4^+^ T cells and CD14^+^ monocytes resulted in increased Treg frequencies (Supplemental Fig. 5A). Additionally, MAPC cells increased Ki67 (Supplemental Fig. 4B) and HLA-DR (Supplemental Fig. 5C) expression on Tregs from co-cultures of both CD45RA^+^ or CD45RO^+^ CD4^+^ T cells with CD14^+^ monocytes. As expected, CD45RA^+^ CD45RO^+^ transitional Tregs were only observed in co-cultures of MAPC cells with CD45RA^+^ CD4^+^ T cells and CD14^+^ monocytes (Supplemental Fig. 5D), further confirming that transitional Tregs emerge from the CD45RA^+^ Tregs and not CD45RO^+^ Tregs.

### MAPC cell induction of Tregs is TGFβ and IDO dependent

**Figure 4:**
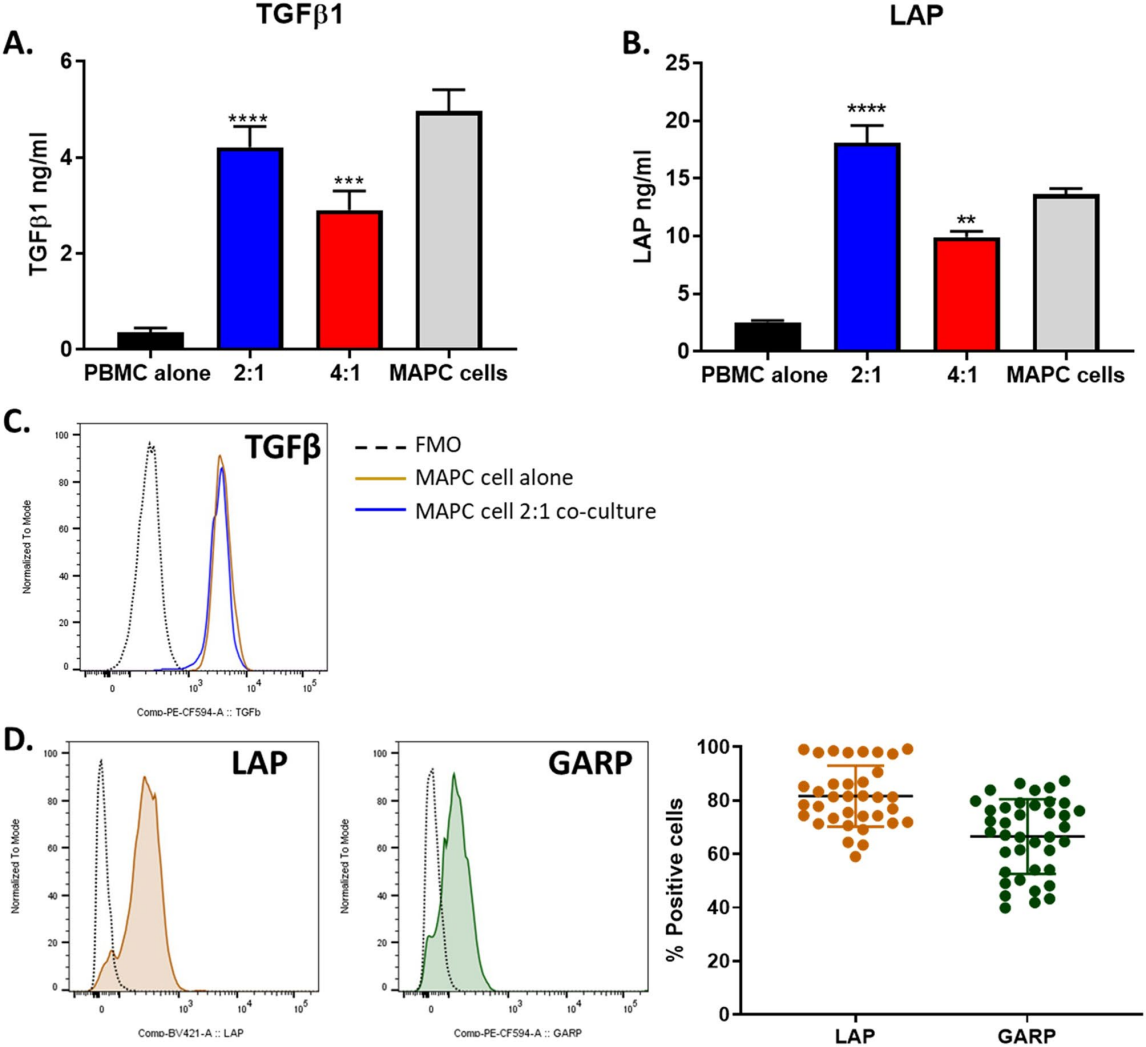
MAPC cells secrete high levels of TGFβ1. (**A**) TGFβ1 and (**B**) LAP concentrations in supernatants from 7-day cultures of PBMC alone (black), 2:1 (blue) and 4:1 (red) PBMC:MAPC cells, and MAPC cells alone (gray) measured by ELISA. MAPC cells were plated alone at 2:1 equivalent cell number. (**C**) Intracellular TGFβ in MAPC cells cultured alone (gold) or with PBMCs at 2:1 PBMC:MAPC cell ratio (blue). FMO represented as dotted line. (**D**) LAP (blue) and GARP (red) surface expression on MAPC cells determined by flow cytometry. FMO shown as dotted black line. Graph showing quantification of LAP (gold) and GARP (green) surface expression on MAPC cells. Data represent mean ± SD from pooled samples of five independent experiments using three different MAPC cell donors. Statistical analysis was performed using One-way ANOVA with a Tukey’s multiple comparisons test reference to the control PBMC alone (****p < 0.0001, ***p < 0.001, and **p < 0.01).

When PBMCs and MAPC cells were co-cultured in Transwells^®^, Treg induction was equivalent to the levels observed when cells were in direct contact, suggesting that MAPC cells promotes Tregs via soluble factors (Supplementary Fig. 3F). TGFβ has been implicated as a strong Treg inducer both in vivo and in vitro^45–48^. First, TGFβ and latent associated protein (LAP) levels within culture supernatants were assessed (Fig. 4A,B). Both TGFβ and LAP levels were elevated within the culture supernatants in the presence of MAPC cells when compared to PBMCs alone. Furthermore, TGFβ and LAP levels were also assessed in supernatants from MAPC cells cultured alone at equal MAPC cell numbers as 2:1 PBMC:MAPC cell ratio. The levels of TGFβ and LAP were equivalent to those seen in the 2:1 PBMC:MAPC cell supernatants, suggesting that MAPC cells are the main source of TGFβ. MAPC cell production of TGFβ was confirmed by intracellular staining of both MAPC cells cultured alone or with PBMCs at 2:1 PBMC:MAPC cell ratio (Fig. 4C). TGFβ production is tightly regulated at a posttranscriptional level^49^. Conversion of latent into mature TGFβ requires cleavage from LAP. Latent TGFβ binds to GARP, a type I transmembrane cell surface docking receptor^50,51^. Since LAP and GARP are associated with TGFβ maturation, the expression of LAP and GARP was assessed on MAPC cells (Fig. 4D). Approximately 80% MAPC cells were positive for LAP, whereas 65% of MAPC cells were GARP positive. Given that supernatants of MAPC cells cultured alone have equivalent levels of TGFβ and LAP as the 2:1 PBMC:MAPC cell co-cultures and that MAPC cells have elevated expression of surface LAP and GARP and intracellular TGFβ imply that MAPC cells are the main source of TGFβ in these cultures.

**Figure 5:**
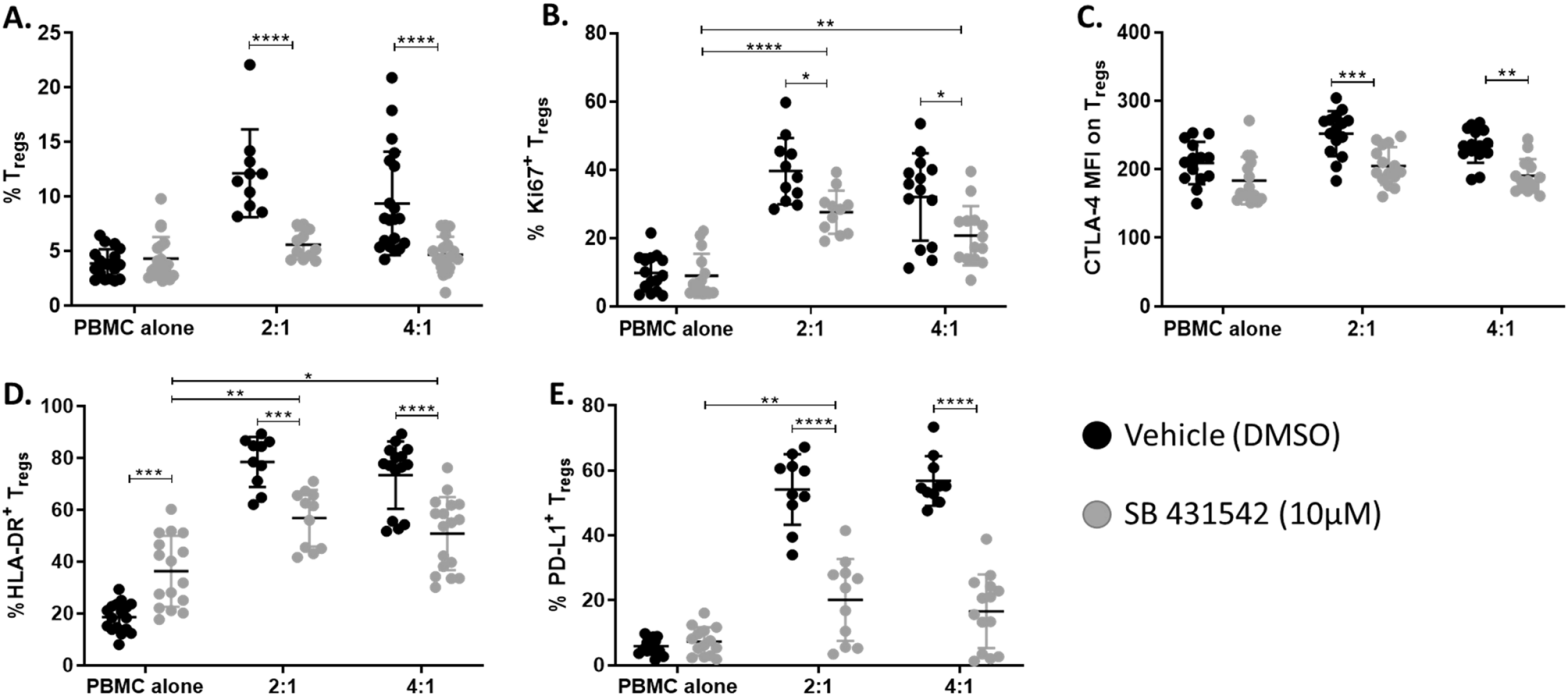
Inhibition of TGFβ signaling decreases MAPC cell mediated induction of Tregs. (**A**) Quantification of Treg percentages after 7-day co-culture with MAPC cells in the presence of vehicle (DMSO, black) or SB 431542 (TGFβR1 kinase inhibitor, grey, 10 μM). Graphs showing quantification of (**B**) Ki67, (**C**) CTLA-4, (**D**) HLA-DR, and (**E**) PD-L1 expression on Tregs. Data represent mean ± SD from pooled samples of five independent experiments using 3 PBMC and 3 MAPC cell donors. Statistical analysis was performed using Two-way ANOVA with a Sidak’s multiple comparisons test (****p < 0.0001, ***p < 0.001, **p < 0.01, and *p < 0.05).

Considering that MAPC cells secrete high levels of TGFβ, the role of TGFβ as a mechanism of action by which MAPC cells induce Tregs was explored. To assess whether TGFβ is involved in MAPC cell-induction of Tregs, PBMCs were co-culture with MAPC cells in the presence of SB 431542, a small molecule TGFβ type 1 receptor (TGFβR1) antagonist (Fig. 5). Blockade of TGFβ signaling completely abrogated MAPC cell induction of Tregs (Fig. 5A), suggesting that TGFβ is a critical component of the mechanism by which MAPC cells promote Tregs. Furthermore, Tregs induced in the presence of SB 431542 had lower Ki67 expression, suggesting less proliferation (Fig. 5B). The abrogation of TGFβ signaling also diminished the expression of CTLA-4, HLA-DR, and PD-L1 on miTregs, further reiterating the important role of TGFβ in the induction of Tregs by MAPC cells (Fig. 5C–E). TGFβ signaling blockade increased HLA-DR expression on PBMC Tregs, while the expression of CTLA-4 and PD-L1 remained unaltered. This could be due to overall increased HLA-DR expression on FoxP3^−^ CD4^+^ cells in the inhibitor treated conditions (Supplemental Fig. 6).

In vitro, MAPC cells inhibit T cell proliferation via the secretion of IDO^14^. Furthermore, IDO has been implicated as an important factor of Treg induction^52,53^. IDO production by MAPC cells was confirmed by intracellular staining of MAPC cells cultured alone or with PBMCs at 2:1 PBMC:MAPC cell ratio (Supplemental Fig. 7A). Analysis of IDO MFI demonstrates that MAPC cells produce similar levels of IDO when cultured alone or with PBMCs. To determine if IDO is involved in MAPC cell induction of Tregs, PBMC and MAPC cells were co-culture in the presence of INCB024360, a selective IDO1 inhibitor. IDO inhibition resulted in a partial reduction of Treg induction by MAPC cells, suggesting that IDO is involved in MAPC cell induction of Tregs, but it is not the primary mechanism (Supplemental Fig. 7B). While Ki67 was still induced in miTregs in the presence of INCB024360, it was reduced when compared to the vehicle treated control (Supplemental Fig. 7C). CTLA-4 induction was not observed in the presence of the IDO inhibitor (Supplemental Fig. 7D). In the presence of IDO blockade, HLA-DR and PD-L1 expression on miTregs were also upregulated. However, HLA-DR was mildly reduced in 2:1 PBMC:MAPC cell condition (Supplemental Fig. 7E), whereas no effect in PD-L1 expression was observed (Supplemental Fig. 7F). Together, these data suggest that IDO is involved in MAPC cell induction of Tregs, however it is not essential.

### CD14^+^ monocytes are involved in MAPC cell induction of Tregs

**Figure 6:**
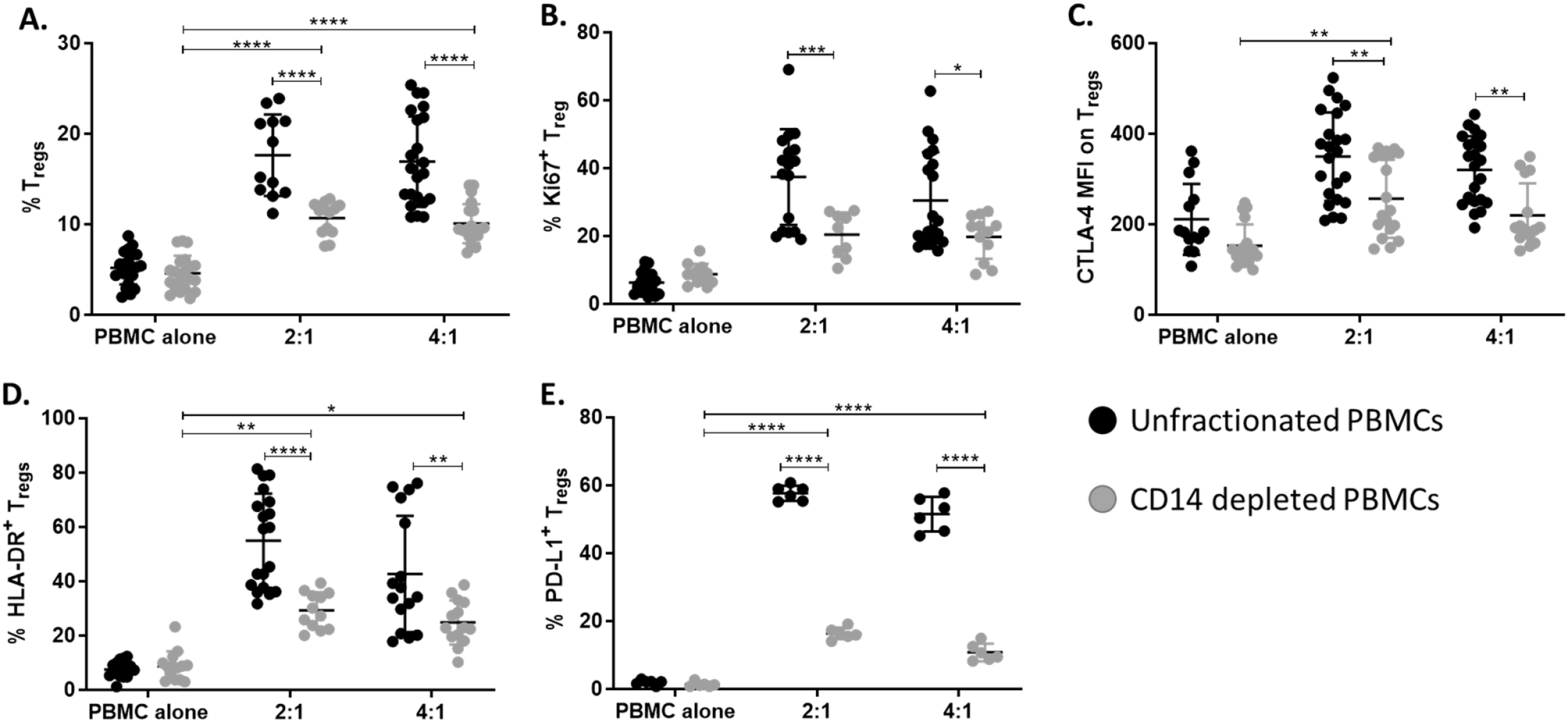
CD14^+^ monocytes are required for optimal MAPC cell induction of Tregs. (**A**) Quantification of Treg percentages after 7-day co-culture of unfractionated PBMCs (black) or CD14 depleted PBMCs (grey) with MAPC cells. Graphs showing quantification of (**B**) Ki67, (**C**) CTLA-4, and (**D**) HLA-DR, and (**E**) PD-L1. Data represent mean ± SD from pooled samples of five independent experiments with 3 PBMC and 3 MAPC cell donors. Statistical analysis was performed using Two-way ANOVA with a Sidak’s multiple comparisons test (****p < 0.0001, ***p < 0.001; **p < 0.01, and *p < 0.05).

MAPC cells and MSC have been shown to modulate myeloid cell responses by skewing their phenotypic profile towards anti-inflammatory cells or “M2”^54–56^. Anti-inflammatory myeloid cells are known to secrete factors such as IL-10, TGFβ, IDO, and retinoic acid that drive Treg differentiation^57–59^ Considering that monocytes can differentiate into dendritic cells and/or macrophages, and in PBMCs, the percentage of dendritic cells is very low (0.3–0.9% of all leukocytes) while monocytes are more abundant (2–12% of leukocytes), the role of monocytes in MAPC cell induction of Tregs was investigated. To determine whether monocytes are indispensable for MAPC cell induction of Tregs, the percentage of Tregs was assessed in MAPC cell co-cultures with PBMCs in which CD14^+^ monocytes were depleted (Fig. 6). In the absence of CD14^+^ monocytes, MAPC cells induced Tregs_,_ but, to a lesser extent than unfractionated PBMCs (Fig. 6A). Interestingly, CD14^+^ monocytes were required for MAPC induction of Ki67 on Tregs (Fig. 6B). Furthermore, while MAPC cells increased the expression of CTLA-4, HLA-DR, and PD-L1 on Tregs induced in the absence of CD14^+^ monocytes, their expression levels were lower than the unfractionated control (Fig. 6C–E). Supernatant levels of TGFβ and LAP remained unaltered suggesting that MAPC is the primary source of TGFβ and LAP in these cultures and not monocytes (Supplementary Fig. 8A,B). These data demonstrate that CD14^+^ monocytes are involved in MAPC cell induction of Tregs.

**Figure 7:**
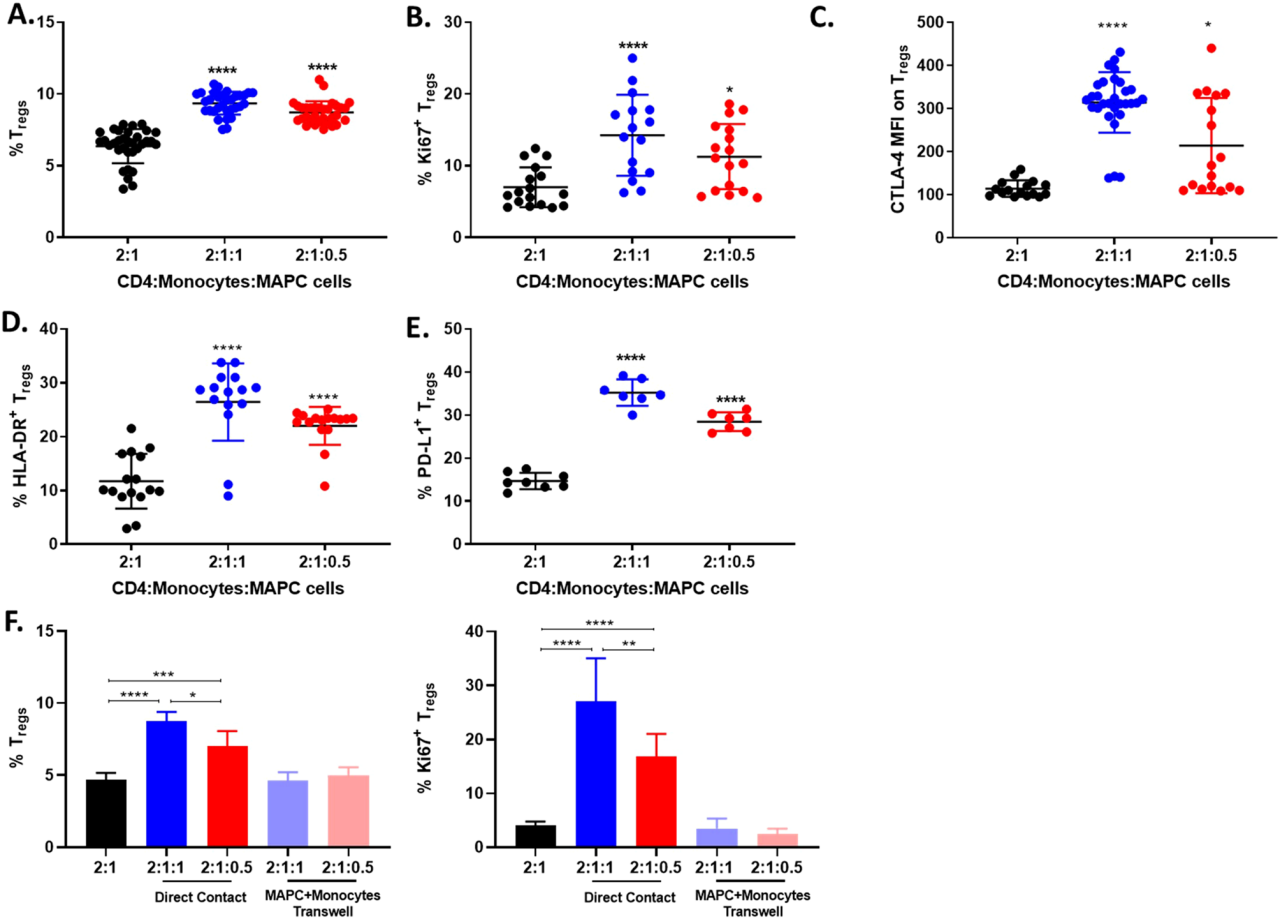
Co-culture of isolated CD4^+^ T cells and CD14^+^ monocytes with MAPC cells induce Treg. (**A**) Quantification of Treg percentages after 7-day co-culture of isolated CD4^+^ T cells and CD14^+^ monocytes (2:1 CD4^+^ T cell:CD14^+^ monocytes) with MAPC cells at 2:1:1 (blue) or 2:1:0.5 (red). Graphs showing quantification of (**B**) Ki67, (**C**) CTLA-4, (**D**) HLA-DR, and **(E**) PD-L1 expression on Tregs**.** Data represent mean ± SD from pooled samples of foue independent experiments with 4 different PBMC donors and 3 MAPC cell donors. Statistical analysis was performed using Two-way ANOVA with a Tukeys’s multiple comparisons test (****p < 0.0001 and *p < 0.05). (**F**) Quantification of Treg percentages and Treg expression of Ki67 after 7-day co-culture of isolated CD4^+^ T cells with CD14^+^ monocytes and MAPC cells at 2:1:1 and 2:1:0.5 in direct contact or with MAPC cells and CD14^+^ monocytes in Transwell. Data represented as mean ± SD from pooled samples of two independent experiments with two different PBMC donors and 2 MAPC cell donors. Statistical analysis was performed using Two-way ANOVA with a Tukeys’s multiple comparisons test (****p < 0.0001, ***p < 0.001; **p < 0.01, and *p < 0.05).

To further confirm the role of CD14^+^ monocytes in MAPC cell induction of Tregs, MAPC cell mediated Treg induction was examined in co-cultures of isolated CD4^+^ T cells and CD14^+^ monocytes (Fig. 7). Interestingly, MAPC cells significantly increased the percentage of Tregs within isolated CD4^+^ T cell and CD14^+^ monocyte co-cultures (Fig. 7A). In addition, miTregs had increased proliferation as shown by their Ki67 expression (Fig. 7B). Furthermore, their CTLA-4, HLA-DR and PD-L1 expression was also increased (Fig. 7C–E). To assess if cell contact between CD4^+^ T cells and CD14^+^ monocytes is required for MAPC cell induction of Tregs, isolated CD4^+^ T cells were cultured in the well while CD14^+^ monocytes were co-cultured with MAPC cells in a Transwell membrane (Fig. 7F). Flow cytometric analysis demonstrated that cell contact between CD4^+^ T cells and CD14^+^ monocytes is indeed required for MAPC induction of Tregs and Treg proliferation. Collectively, these data demonstrate that the mechanism by which MAPC cells induce Tregs and promote their proliferation is dependent on CD14^+^ monocytes.

**Figure 8:**
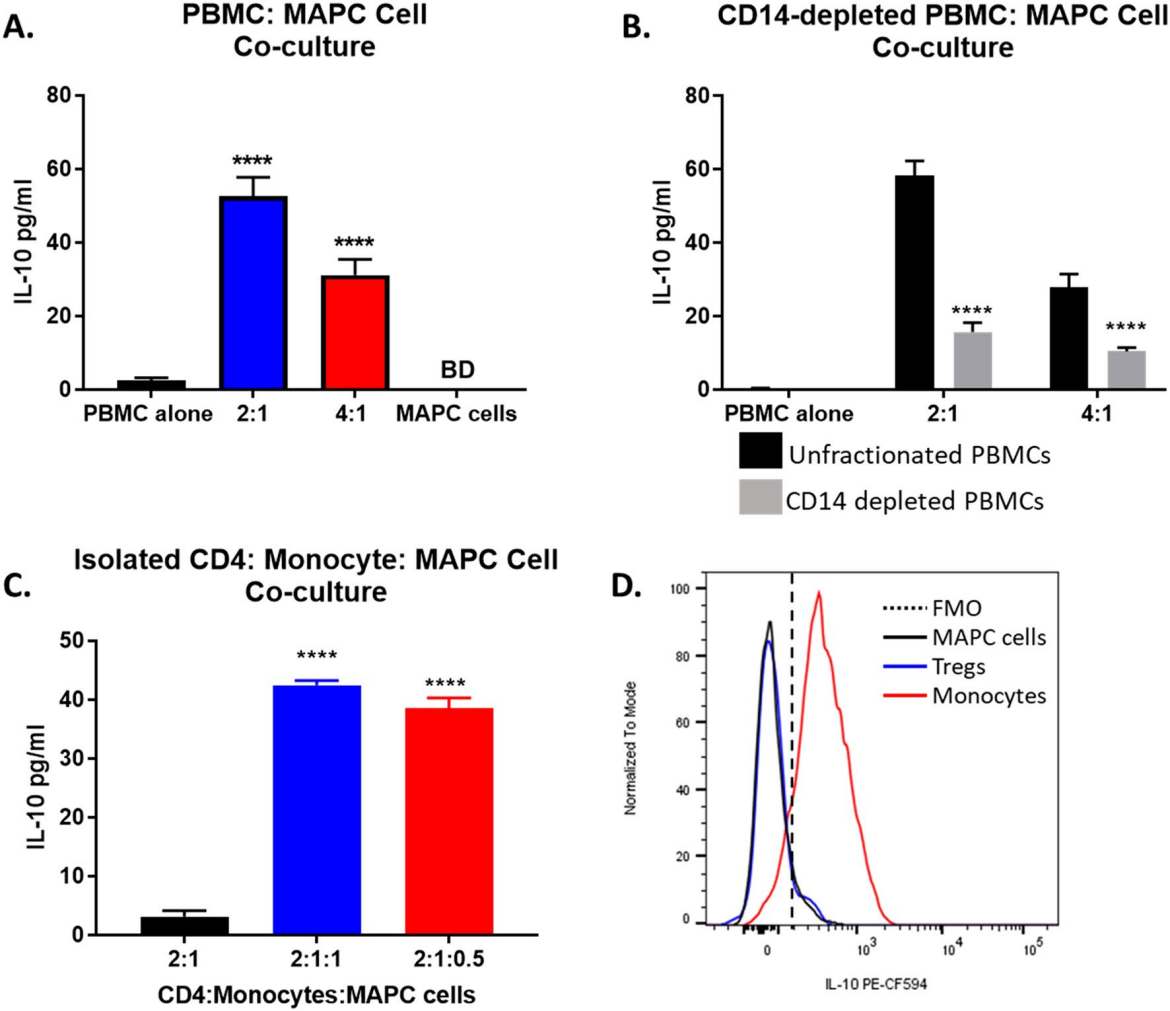
MAPC cells promotes monocyte secretion of IL-10. (**A**) IL-10 concentration in cultures supernatants of PBMC alone (black), 2:1 (blue) and 4:1 (red) PBMC:MAPC cells, and MAPC cells alone (gray) (BD, below detection) measured by ELISA. (**B**) IL-10 levels in supernatants collected from PBMCs (black) or CD14 depleted PBMCs (gray) cultured alone or with MAPC at 2:1 and 4:1 PBMC:MAPC ratios for 7 days measured by ELISA. Statistical analysis was performed using Two-way ANOVA with a Sidak’s multiple comparison test (****p < 0.0001) (**C**) IL-10 concentration in supernatants from cultures of isolated CD4^+^ T cells and CD14^+^ monocytes (2:1 CD4^+^ cell:CD14^+^ monocytes) with MAPC cells at 2:1:1 (blue) or 2:1:0.5 (red) measured by ELISA. Data represent mean ± SD from pooled samples of six independent experiments. Statistical analysis was performed using Two-way ANOVA with a Tukeys’s multiple comparisons test (****p < 0.0001). (**D**) Representative histogram depicting intracellular IL-10 expression on MAPC cells (black line), Tregs (blue), and monocytes (red). FMO control shown as dotted line.

IL-10 is a potent anti-inflammatory cytokine secreted by several immune cells including Tregs, monocytes, macrophages, and dendritic cells. To assess whether MAPC cells affect IL-10 secretion, IL-10 levels in PBMC:MAPC cell co-culture supernatants were measured by ELISA (Fig. 8A). Minimal IL-10 levels were found in supernatants from PBMCs and MAPC cells cultured alone. Interestingly, PBMCs and MAPC cell co-cultures had elevated IL-10 concentration. Analysis of supernatants collected from CD14-depleted: MAPC cell co-cultures demonstrated that IL-10 levels were reduced in the absence of CD14^+^ monocytes (Fig. 8B). Conversely, IL-10 levels on co-cultures of isolated CD4^+^ T cells, monocytes, and MAPC cells were comparable to those observed in PBMC:MAPC co-cultures (Fig. 8C), suggesting that the source of IL-10 is either CD4^+^ T cells or monocytes. To identify the cell responsible for IL-10 production, PBMC:MAPC cells were co-cultured for 7 days. At day 7, GolgiStop was added to the cultures overnight, and IL-10 was measured intracellularly by flow cytometry. IL-10^+^ cells were found to be CD14^+^ monocytes and not CD4^+^ T cells nor MAPC cells (Fig. 8D), confirming monocytes as the primary producer of IL-10 in these cultures. Backgating analysis of IL-10^+^ cells demonstrated that most of the IL-10^+^ cells were CD14^+^ monocytes.

## Discussion

Like MSCs, MAPC cells have been shown to modulate inflammation in multiple models of injury and disease^11,12,14–19,25,60^. MAPC cells control immune responses by secreting a milieu of factors with immunomodulatory properties, thus, restoring the inflammatory balance and promoting tissue repair^14–16,61^. Furthermore, MAPC cells have been demonstrated to increase the frequency of Tregs which are widely known for their capacity to control immune responses and induce tolerance. MSC induction of Tregs has been widely studied^62–67^, whereas little is known about the mechanisms involved in MAPC cell induction of Tregs. Herein, as illustrated on Supplemental Fig. 8, we describe the mechanisms by which MAPC cells induce Tregs and characterize the phenotype of miTregs. While the studies shown herein were conducted under non-inflammatory conditions to avoid transient expression of FoxP3 on activated CD4^+^ T cells, we provide compelling evidence of potential mechanisms involved in the MAPC cell mediated induction of Tregs that are known to be involved in in vivo induction of Tregs in other models.

First, we observed that MAPC cells consistently increased the frequency and total cell numbers of Tregs in a dose-dependent fashion. miTregs expressed higher levels of Foxp3 and CD25 per cell than PBMC Tregs, suggesting that miTregs may be more potent immune regulators than PBMC Tregs, as elevated levels of FoxP3 Tregs have been linked with lower transplant rejection, increased suppressive activity, and higher secretion of IL-10 and TGFβ in a murine model of orthotropic corneal transplantation^32^. Conversely, Tregs expressing low levels of CD25 have been associated with autoimmunity^33^. Also, in mice, upregulation of CD25 expression on Tregs correlates with enhanced Treg function^34^.

Co-cultures of MAPC cells with CD25-depleted PBMCs demonstrated that MAPC cells promote the differentiation of CD4^+^ T cells into Tregs. Moreover, in the presence of MAPC cells, Tregs had increased proliferation, suggesting that MAPC cells also increase the frequency of Tregs by stimulating their expansion. Conversely, MAPC induced proliferation was restricted to Tregs and not FoxP3^−^ CD4^+^ T cells, as previously demonstrated in an in vitro model of homeostatic proliferation^14^. MAPC cells induced Tregs to produce low levels of IL-2, cytokine involved in Treg survival, proliferation, and function^68,69^. Given that IL-2 is a strong inducer of Treg proliferation, it is possible that IL-2 secreted by miTregs is involved in the induction of Treg proliferation seen in PBMC:MAPC cell co-cultures.

Phenotypical analysis of cell surface markers demonstrated that miTregs express higher levels of CTLA-4, HLA-DR, and PD-L1 than PBMC Tregs. CTLA-4 is a potent regulator of T cell activation by competing with CD28 for the binding of the B7 costimulatory molecules CD80 and CD86^34^. In humans, Tregs expressing high levels of CTLA-4 have increased suppressive capacity than CTLA-4 low expressing counterparts^37,46^. HLA-DR^+^ Tregs express higher levels of FoxP3^35^. In vitro, activation of HLA-DR^−^ Tregs induces HLA-DR expression^35,43,70^. Parallel comparison of HLA-DR^+^ versus HLA-DR^−^ Tregs demonstrated that HLA-DR^+^ Tregs are more efficient suppressors and HLA-DR expression defines a terminally differentiated Treg^34,35^. MAPC cell mediated induction of HLA-DR was restricted to the Treg population and no other cell types, suggesting that MAPC cells preferentially activate Tregs. Ligation of PD-1 with PD-L1 sends inhibitory signals, thereby downregulating immune activation. Tregs from PD-L1^−/−^ mice have been shown to have impaired suppressive capacity in vitro and in an in vivo model of nephrotoxic nephritis, suggesting that PD-L1 expression on Tregs is important for Treg suppressive function^71^. Moreover, stimulation of CD4^+^ T cells with anti-CD3/CD28 in the presence of recombinant PD-L1 increases Tregs by promoting CD4^+^ T cell differentiation into Tregs and driving Treg expansion^72^. Thus, the increased expression of CTLA-4, HLA-DR, and PD-L1 in miTregs further supports that MAPC cells induce a potent suppressive phenotype on Tregs. Furthermore, miTregs secreted higher levels of TGFβ than Tregs from PBMCs, also suggesting an enhanced suppressive capacity. Using a T cell suppression assay, it was confirmed that miTreg are indeed significantly more suppressive of T cell proliferation when compared to PBMC Tregs. While MAPC cell induction of Tregs has been described in vivo^17,18,24,26^, phenotypic characterization of Tregs induced by MAPC cells in vivo remains to be evaluated.

The CD45 isoforms CD45RA and CD45RO are used to identify resting (CD45RA^+^ FoxP3^+^ CD25^+^) versus activated (CD45RO^+^ FoxP3^+^ CD25^+^) Tregs^41,44^. Our studies demonstrated that MAPC cells increase the frequency of a transitional/recently activated Treg expressing both CD45RA and CD45RO. The transitional/recently activated Tregs arose from the CD45RA^+^ Treg population and not from CD45RO^+^ Tregs. Detailed analysis of the phenotype of this transitional population provided evidence that these cells express higher levels of Ki67, correlating with an active proliferative state along with increased CTLA-4 and HLA-DR than the CD45RA^+^ and CD45RO^+^ counterparts, consistent with a recently activated phenotype. MAPC cells also increased the expression of Ki67, CTLA-4, and HLA-DR on both CD45RA^+^ and CD45RO^+^ Tregs; however, consistent with an activated state, CD45RO^+^ Tregs have higher expression of these markers than CD45RA^+^ Tregs.

MAPC cells secrete a variety of paracrine factors that can modulate the immune system including TGFβ and IDO^14,73–75^. The results of our studies demonstrate that TGFβ secreted by MAPC cells is a primary mechanism by which MAPC cells induce Tregs. Blockade of TGFβ signaling using SB 431542, a TGFβ receptor antagonist, significantly abrogated MAPC cell induction of Tregs. In the absence of TGFβ signaling, MAPC cells were less efficient at promoting Treg proliferation and expression of suppressive markers. In addition to TGFβ being a primary driver of MAPC cell induction of Tregs, IDO was also identified as a contributing factor. The inhibition of IDO, another factor secreted by MAPC cells, partially reduced Treg induction supporting IDO involvement in Treg induction by MAPC cells. Both TGFβ and IDO have been extensively identified as key factors secreted by tolerogenic dendritic cells that promote Treg differentiation^45,48,52,53,57–59^. Indeed, TGFβ and IDO have been proposed as potential mechanisms by which MSCs induces Treg differentiation^76,77^. Herein, we demonstrate that these factors also play an important role in MAPC cell mediated Treg induction.

The role of monocytes in MAPC cell induction of Tregs was also explored. Monocytes differentiate into dendritic cells and macrophages and secrete a variety of factors known to promote Tregs. Depletion of CD14^+^ monocytes demonstrated that these cells are involved in MAPC cell induction of Treg proliferation and expression of CTLA-4, PD-L1, and HLA-DR. In fact, in the absence of CD14^+^ monocytes, MAPC cells were not able to induce Treg proliferation. These observations were confirmed by co-culture of isolated CD4^+^ T cells, CD14^+^ monocytes, and MAPC cells. In this setting, MAPC cells supported Treg induction by increasing their proliferation and expression of a suppressive phenotype. Transwell experiments revealed that direct contact between CD4^+^ T cells and CD14^+^ monocytes is necessary for MAPC cell induction of Tregs and Treg proliferation. It is possible that CD14^+^ monocytes are providing T cell receptor (TCR) stimulation and co-stimulatory signals to Tregs, driving their proliferation. Additional experiments are required to discern the contribution of monocytes in MAPC cell induction of Tregs. The involvement of monocytes in the induction of Tregs by MSCs has been previously described^67,78^. Researchers demonstrated that MSCs skew monocytes towards an anti-inflammatory phenotype, thereby facilitating Treg differentiation^78^. MAPC cells have also been demonstrated to induce a comparable anti-inflammatory phenotype on myeloid cells in vitro^55^ and in vivo^18^, however, the effect of MAPC cells on CD14^+^ monocytes responsible for their involvement in MAPC-mediated induction of Tregs remains to be elucidated.

MAPC cells also increased the IL-10 levels in the PBMC:MAPC cell co-culture supernatants. Lower IL-10 levels were observed in supernatants from monocyte-depleted PBMC:MAPC cell co-cultures, suggesting that monocytes might be the primary source of IL-10. Flow cytometric analysis demonstrated that monocytes were indeed the primary source of IL-10. Unlike MSCs^79,80^, MAPC cells did not produce IL-10 when co-cultured with PBMC. IL-10 has been shown to enhance TGFβ-induced Treg differentiation and suppressive capacity via STAT3 and Foxo1, suggesting that monocyte derived IL-10 might be involved in MAPC induction of Tregs^81^.

MAPC cells have potent immunoregulatory properties under a variety of conditions including homeostatic proliferation, graft vs host disease, spinal cord injury, traumatic brain injury and ischemic stroke^14–19,61^. Herein, we provide evidence demonstrating that MAPC cells induce Tregs, cells known to play key roles in modulating immune responses in vitro and in vivo, via various mechanisms involving the secretion of TGFβ, IDO, and CD14^+^ monocytes. Furthermore, characterization of miTregs reveals a more potent immunomodulatory phenotype and highlights a mechanistic pathway where MAPC cells may modulate immune responses under different clinical conditions. Further investigation will be performed in vivo to examine the mechanisms identified in this study involved in MAPC induction of Tregs and perform a functional and phenotypical assessment of miTregs.

## Methods

### Cell culture

MAPC cells were generated from donor’s bone marrow aspirate as previously described^14,82^. Informed consent was obtained in accordance with the guidelines of a commercial Institutional Review Board for all healthy donors of MAPC cells. Prior to use, MAPC cell phenotype was assessed by flow cytometry. As previously described, all MAPC cells used were over 90% positive for CD49c and CD90, whereas < 5% of the cells expressed HLA-DR and CD45^14^, thereby confirming that the MAPC cells used were in fact a homogenous population. Three different MAPC cell donors were used in this study. The population doublings for all MAPC cells used ranged from 20 to 35.

Human subjects’ research approval was obtained from Western Institutional Review Board, Inc. (Puyallup, WA) and written informed consent was obtained from all healthy volunteers involved in this study. All experiments and methods were conducted in accordance with Western Institutional Review Board relevant guidelines and regulations. Peripheral blood mononuclear cells (PBMCs) were isolated from fresh blood of healthy volunteers (ten different donors) using Ficoll-Paque (GE Healthcare Life Sciences, Pittsburgh, PA) density gradient centrifugation as indicated by the manufacturer. Alternatively, isolated frozen PBMCs from healthy donors (two different donors) were purchased from Precision for Medicine (Frederick, MD) and PPA Research (Johnson City, TN). PBMCs (1 × 10^6^) were co-cultured with MAPC cells at different ratios in RPMI 1640 media supplemented with 10% heat inactivated FBS and 1% penicillin/streptomycin in a 24-well plate. At day 7 post co-culture, supernatants were collected and the percentage of Tregs was determined by flow cytometry.

Alternatively, CD14^+^ monocytes were depleted from PBMCs using anti-CD14 microbeads (Miltenyi Biotec, Auburn, CA) following the manufacturer’s instructions. Similarly, CD14^+^ monocytes were isolated by positive selection using anti-CD14 microbeads (Miltenyi Biotec), whereas CD4^+^ T cells were isolated by negative selection using Dynabeads Untouched Human CD4 T cells kit (ThermoFisher, Waltham, MA). The purity of each cell type isolated was determined by staining cells with anti-CD14 (M5E2) PE, anti-CD3 (UCHT1) APC, and anti-CD4 (SK3) PerCpCy5.5 (BD Biosciences, San Diego, CA) as described below. Cell purity was over 90% for each isolated population. Isolated cells were plated as follows: 0.5 × 10^6^ CD4^+^ T cells and 0.25 × 10^6^ CD14^+^ monocytes (2:1); 0.5 × 10^6^ CD4^+^ T cells, 0.25 × 10^6^ CD14^+^ monocytes, and 0.25 × 10^6^ MAPC cells (2:1:1); 0.5 × 10^6^ CD4^+^ T cells, 0.25 × 10^6^ CD14^+^ monocytes, and 0.125 × 10^6^ MAPC cells (2:1:0.5).

CD25 positive cells were depleted using CD25 Microbeads (Miltenyi) following manufacturer’s instructions. Approximately 70% of Tregs were depleted by CD25 Microbeads (Supplemental Fig. 3A). To enrich the CD45RA and CD45RO CD4^+^ T cell populations, CD4^+^ T cells were isolated using Dynabeads Untouched Human CD4 T cells kit followed by a positive selection of CD45RO^+^ cells using CD45RO microbeads (Miltenyi Biotec) following manufacturer’s instructions. Cells collected from the flow through were enriched for CD45RA. Cell purity was over 88% and 65% for CD45RO and CD45RA CD4^+^ T cells, respectively.

### Flow cytometry

After supernatant collection, cells were washed with ice cold FACS buffer (2% heat inactivated FBS 2 mM EDTA in D-PBS) for 5 min at 450 × *g* on high brake. Cells were stained with BD Horizon Fixable Viability stain 780 (BD Biosciences) following manufacturer’s instructions. After viability staining, cells were stained for surface markers using the following antibodies: anti-CD4 (RPA-T4) Pacific Blue, anti-CD3 (UCHT1) PerCP Cy5.5, anti-CD25 (2A3) PE, anti-HLA-DR (G46-6) BV650, and anti-PD-L1 (MIH1) BV786 (BD Biosciences) for 30 min on ice protected from light. Alternatively, to determine CD45RA and CD45RO expression, cells were stained with anti-CD4 Pacific Blue, anti-CD3 PerCP Cy5.5, anti-CD25 PE, anti-HLA-DR BV650, anti-CD45RA (HI100) Alexa Fluor 700, and anti-CD45RO (UCHL1) BV605 (BD Biosciences).

After staining cell surface markers, cells were washed and fixed/permeabilized using FoxP3/Transcription Factor Staining Buffer Set (ThermoFisher) as directed by the manufacturer. Subsequent to the fixation/permeabilization, cells were stained using the following antibodies: anti-FoxP3 (259D/C7) Alexa Fluor 647, anti-Ki67 (B56) BV510 (BD Biosciences), and anti-CTLA-4 (14D3) FITC (ThermoFisher).

To identify Tregs, cells were first gated based on lymphocyte size and granularity using forward and side scatter (FSC and SSC), followed by gating on single cells using FSC area versus FSC height. Live cells were selected based on the absence of Fixable Viability stain 780. CD4^+^ T cells were identified by their expression of CD3 and CD4. Tregs were identified as CD3^+^ CD4^+^ FoxP3^+^ CD25^+^ (Supplemental Fig. 1). Since CD4^−^ T cells are known to have low expression of Foxp3^83^, CD4^−^ T cells were used as a negative control for Foxp3 (Supplemental Fig. 1E).

To determine the expression of latent associated peptide (LAP) and glycoprotein-A repetitions predominant (GARP) on MAPC cells, MAPC cells were cultured for 7 days in RPMI media supplemented with 10% heat inactivated FBS and 1% penicillin/streptomycin. Cells were washed and stained for the surface expression of LAP and GARP using the following antibodies: anti-LAP (TW4-9E7) BV421 and anti-GARP (7B11) PE-CF594 (BD Biosciences), as described above. Alternatively, LAP and GARP expression was determined on MAPC out of thaw.

To identify the primary source of IL-10, PBMCs were cultured with or without MAPC cells for 7 days in RPMI media supplemented with 10% heat inactivated FBS and 1% penicillin/streptomycin. GolgiStop (BD Biosciences) was added to cultures according to manufacturer’s instructions (0.67 µL of GolgiStop per mL of media) overnight (~ 16–18 h). Cells were stained with Fixable Viability Stain 780 to exclude dead cells, followed by surface staining with anti-CD14 Pacific Blue, anti-CD3 PerCP Cy5.5, anti-CD25 PE, and anti-CD127 (HIL-7R-M21) Alexa Fluor 647 as described above. Cells were permeabilized using FoxP3/Transcription Factor Staining Buffer Set as described above and stained with anti-IL-10 (JES3-19F1) PE-CF594. Intracellular IL-10 was assessed in CD14^+^ monocytes, CD25^+^ CD127^low^ CD4^+^ Tregs, and MAPC cells. Alternatively, to identify the source of TGFβ and IL-2 cells were stained with anti-TGFβ (TW4-9E7) PE-CF594, anti-IDO (eyedio) FITC or anti-IL-2 (MQ1-17H12) PE.

All samples were analyzed using a BD FACSCelesta flow cytometer equipped with a blue, violet, and red laser configuration. Data collected from these experiments were analyzed using FlowJo software (Ashland, Oregon). Spectral spillover and fluorescent compensation were generated using BDCompBeads sets containing polystyrene beads coupled with antibody specific to mouse, rat, or hamster Ig, κ light chain and negative control beads (BD Biosciences). Positive expression was based on fluorescent minus one (FMO) controls.

### T cell suppression assay

To assess Treg suppressive function, isolated PBMCs were cultured alone or with MAPC cells at a 2:1 PBMC:MAPC cell ratio for 7 days as described above. At day 7, Tregs were isolated using EasySep Human CD4 + CD127LowCD25 + Regulatory T cell isolation kit (STEMCELL Technologies, Cambridge, MA) following manufacturer’s instructions. Over 85% of the cells isolated were Tregs based on their phenotypic profile (CD3^+^ CD4^+^ CD25^+^, FoxP3^+^, and CD127^low^) assessed by flow cytometry. Isolated Tregs were co-cultured with CellTrace Violet stain (ThermoFisher) labeled autologous PBMCs stimulated with anti-CD3/CD28 Dynabeads (1 × 10^5^ beads/ml, ThermoFisher) in RPMI 1640 media supplemented with 10% heat inactivated FBS and 1% penicillin/streptavidin. At day 5 post stimulation, cells were stained with BD Horizon Fixable Viability stain 780, anti-CD3 PerCP Cy5.5, and anti-CD25 PE (BD Biosciences). Samples were analyzed by flow cytometry using BD FACSCelesta. Tregs were excluded based on their CD25^hi^ expression and lack of CellTrace Violet stain. T cell proliferation was assessed by measuring CellTrace Violet stain dilution within the CD3^+^ T cell population. Percent suppression was calculated using the following equation:

$$\% \;{\text{Suppression}} = \frac{{{\text{Proliferated}}\;{\text{CD}}3\;{\text{Positive}}\;{\text{control}} - {\text{Proliferated}}\;{\text{CD}}3\;{\text{in}}\;{\text{Treg}}\;{\text{treated}}\;{\text{sample}}}}{{{\text{Proliferated}}\;{\text{CD}}3\;{\text{Positive}}\;{\text{control}}}} \times 100.$$

### TGFβ and IDO inhibition experiments

To inhibit TGFβ signaling, PBMCs were co-cultured with MAPC cells in the presence of SB 431542 (Tocris, Minneapolis, MN), a selective inhibitor of TGFβ receptor 1 kinase (TGFβR1) at a concentration of 10 μM^84^. SB 431542 was dissolved in dimethyl sulfoxide (DMSO) at 50 mM as indicated by the manufacturer. Vehicle controls were treated with equal amounts of DMSO.

IDO was inhibited using INCB024360, a potent and selective IDO1 inhibitor (Cayman Chemical, Ann Arbor, MI) added to PBMC:MAPC cell co-cultures at a concentration of 10 μM^85^. INCB024360 was dissolved in DMSO at 0.11 M following manufacturer’s guidelines. Vehicle controls received equal amounts of DMSO. Treg induction was determined by flow cytometry as described above.

### ELISA

The levels of TGFβ, LAP, IL-2, and IL-10 in culture supernatants were measured using human Quantikine ELISA kits from R&D Systems (Minneapolis, MN) following manufacturer’s instructions.

### Statistical analysis

Data represent the mean ± SD as indicated in figure legends. P-values were determined by One-way ANOVA with a Tukey’s multiple comparison test, Student’s *t* Test, or Two-way ANOVA with Sidak’s multiple comparison test using GraphPad Prism 8.4.2 (San Diego, CA) as indicated in figure legends.

### Ethics approval

The use of healthy donor approval was obtained from Western Institutional Review Board, Inc. (Puyallup, WA) and informed consent was obtained from healthy volunteers as appropriate.

## Supplementary Information

Supplementary Information.

## Data Availability

The data supporting the findings of this study are available from the corresponding author upon reasonable request.

## Abbreviations

MAPC cells: Multipotent adult progenitor cells
AMI: Acute myocardial infarction
GvHD: Graft versus host disease
ARDS: Acute respiratory distress syndrome
Tregs: Regulatory T cells
PBMCs: Peripheral blood mononuclear cells
miTregs: MAPC cell-induced Tregs
TGFβ: Transforming growth factor beta
IDO: Indoleamine 2,3 dioxygenase
CTLA-4: Cytotoxic T lymphocyte associated protein 4
PD-L1: Programmed death ligand 1
HLA-DR: Human leukocyte antigen DR isotype
nTregs: Natural Tregs
iTregs: Induced Tregs
CD: Cluster of differentiation
IPEX: Immunodysregulation polyendocrinopathy enteropathy X-linked
IL-10: Interleukin 10
IL-35: Interleukin 35
cAMP: Cyclic adenosine monophosphate
IL-2: Interleukin 2
MLR: Mixed lymphocyte reaction
PGE-2: Prostaglandin E2
FBS: Fetal bovine serum
DMSO: Dimethyl sulfoxide
LAP: Latent associated protein
GARP: Glycoprotein-A repetitions predominant
MFI: Median fluorescent intensity
MSC: Mesenchymal stem cells
