# Supplementary material for: Multipotent adult progenitor cells induce regulatory T cells and promote their suppressive phenotype via TGFβ and monocyte-dependent mechanisms
Source: Sci Rep. 2021 Jun 30;11:13549. doi: 10.1038/s41598-021-93025-x (PMC8245558; doi:10.1038/s41598-021-93025-x)
Supplement: Supplementary file 1 — Supplementary Information. [file 41598_2021_93025_MOESM1_ESM.pptx]

## Slide 1
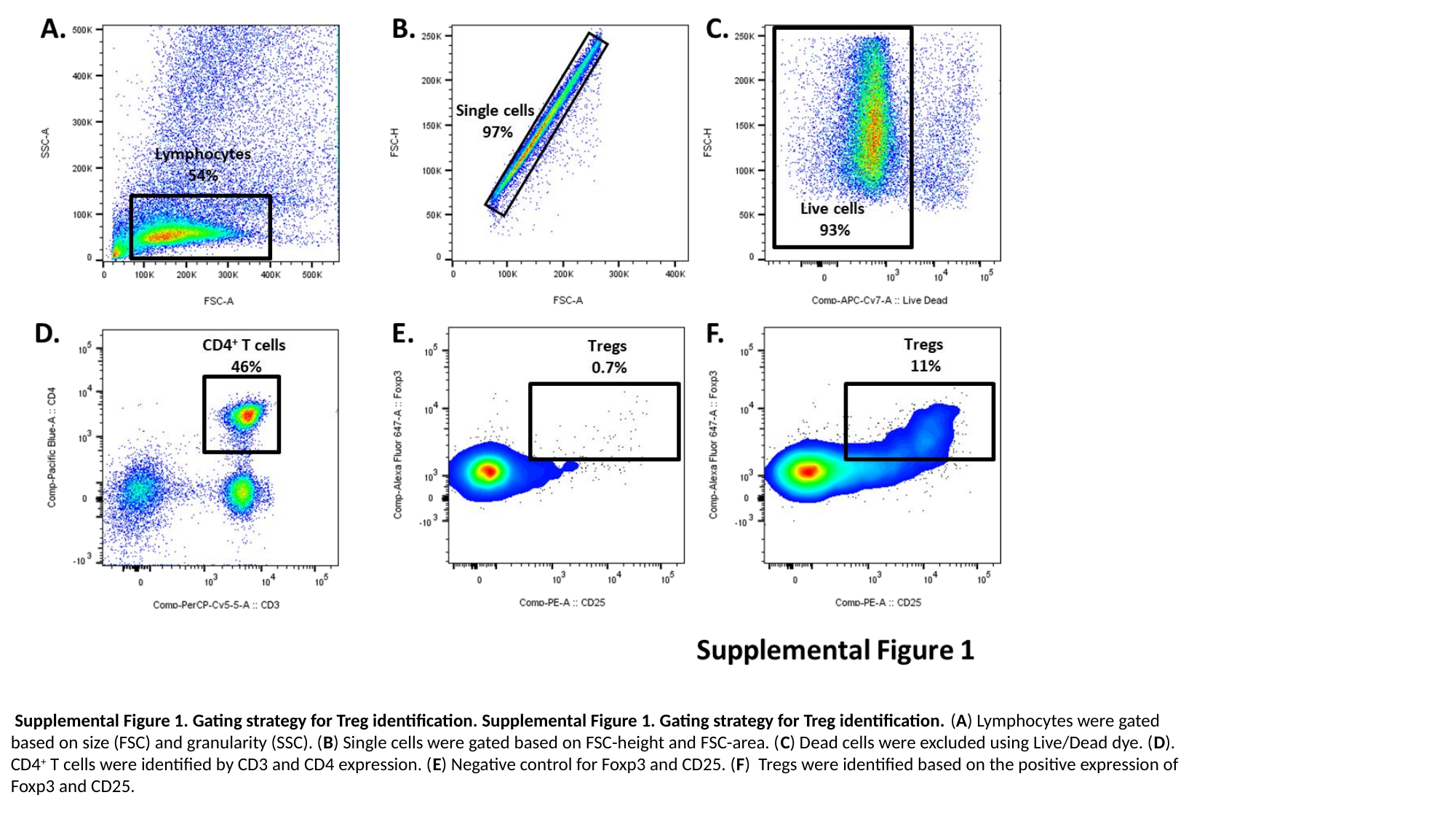

Supplemental Figure 1. Gating strategy for Treg identification. Supplemental Figure 1. Gating strategy for Treg identification. (A) Lymphocytes were gated based on size (FSC) and granularity (SSC). (B) Single cells were gated based on FSC-height and FSC-area. (C) Dead cells were excluded using Live/Dead dye. (D). CD4+ T cells were identified by CD3 and CD4 expression. (E) Negative control for Foxp3 and CD25. (F) Tregs were identified based on the positive expression of Foxp3 and CD25.

## Slide 2
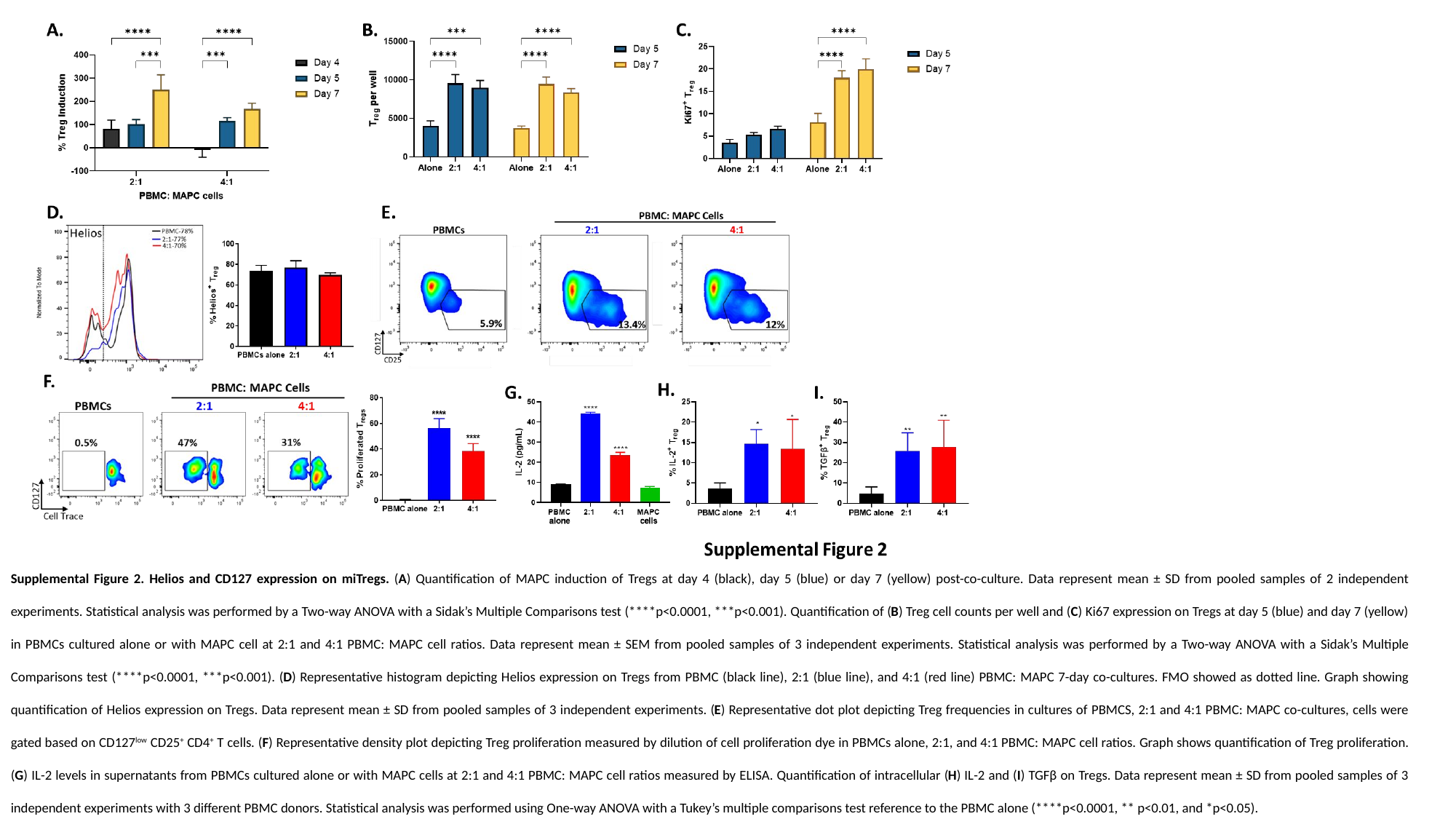

Supplemental Figure 2. Helios and CD127 expression on miTregs. (A) Quantification of MAPC induction of Tregs at day 4 (black), day 5 (blue) or day 7 (yellow) post-co-culture. Data represent mean ± SD from pooled samples of 2 independent experiments. Statistical analysis was performed by a Two-way ANOVA with a Sidak’s Multiple Comparisons test (****p<0.0001, ***p<0.001). Quantification of (B) Treg cell counts per well and (C) Ki67 expression on Tregs at day 5 (blue) and day 7 (yellow) in PBMCs cultured alone or with MAPC cell at 2:1 and 4:1 PBMC: MAPC cell ratios. Data represent mean ± SEM from pooled samples of 3 independent experiments. Statistical analysis was performed by a Two-way ANOVA with a Sidak’s Multiple Comparisons test (****p<0.0001, ***p<0.001). (D) Representative histogram depicting Helios expression on Tregs from PBMC (black line), 2:1 (blue line), and 4:1 (red line) PBMC: MAPC 7-day co-cultures. FMO showed as dotted line. Graph showing quantification of Helios expression on Tregs. Data represent mean ± SD from pooled samples of 3 independent experiments. (E) Representative dot plot depicting Treg frequencies in cultures of PBMCS, 2:1 and 4:1 PBMC: MAPC co-cultures, cells were gated based on CD127low CD25+ CD4+ T cells. (F) Representative density plot depicting Treg proliferation measured by dilution of cell proliferation dye in PBMCs alone, 2:1, and 4:1 PBMC: MAPC cell ratios. Graph shows quantification of Treg proliferation. (G) IL-2 levels in supernatants from PBMCs cultured alone or with MAPC cells at 2:1 and 4:1 PBMC: MAPC cell ratios measured by ELISA. Quantification of intracellular (H) IL-2 and (I) TGFβ on Tregs. Data represent mean ± SD from pooled samples of 3 independent experiments with 3 different PBMC donors. Statistical analysis was performed using One-way ANOVA with a Tukey’s multiple comparisons test reference to the PBMC alone (****p<0.0001, ** p<0.01, and *p<0.05).

## Slide 3
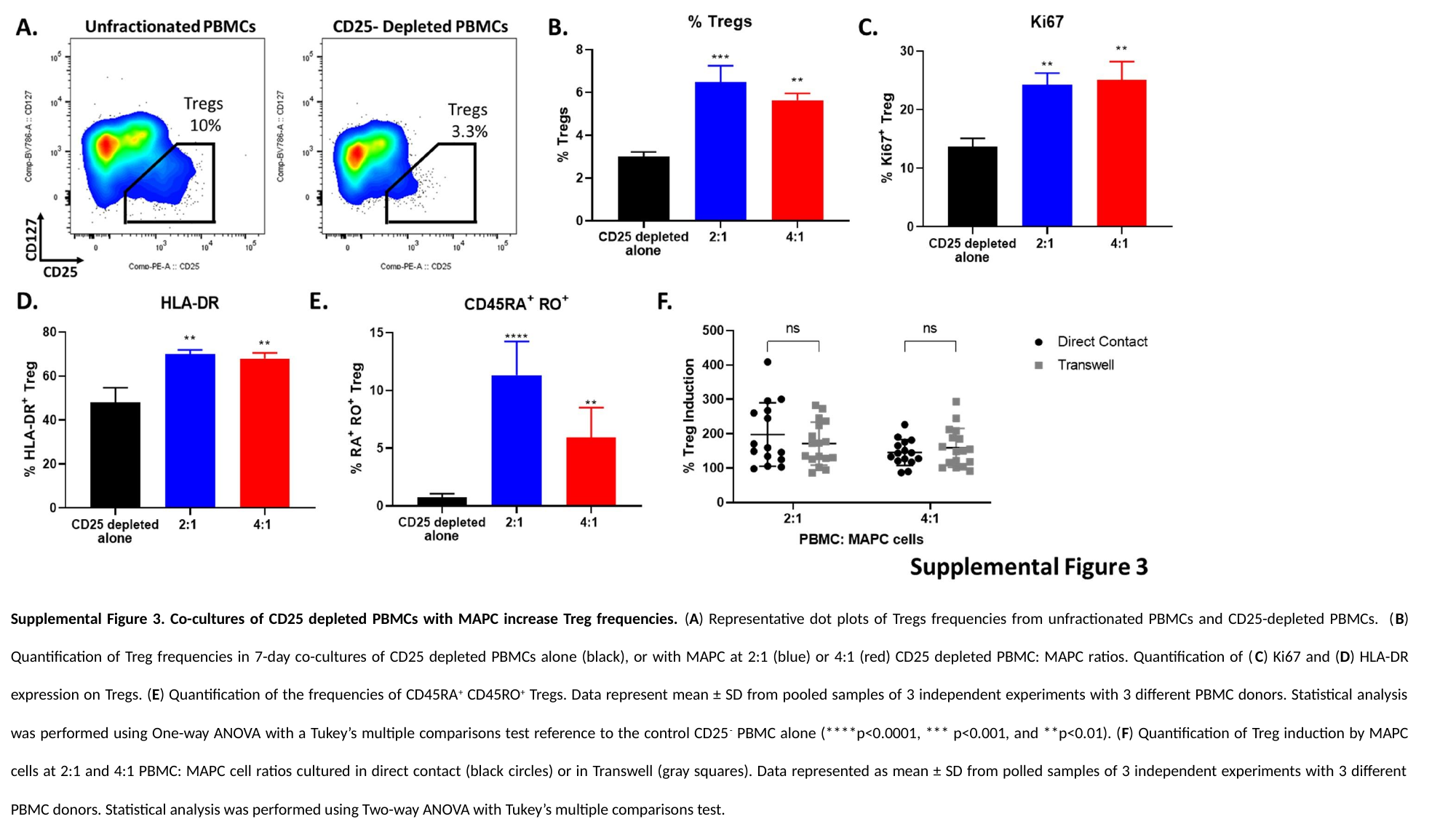

Supplemental Figure 3. Co-cultures of CD25 depleted PBMCs with MAPC increase Treg frequencies. (A) Representative dot plots of Tregs frequencies from unfractionated PBMCs and CD25-depleted PBMCs. (B) Quantification of Treg frequencies in 7-day co-cultures of CD25 depleted PBMCs alone (black), or with MAPC at 2:1 (blue) or 4:1 (red) CD25 depleted PBMC: MAPC ratios. Quantification of (C) Ki67 and (D) HLA-DR expression on Tregs. (E) Quantification of the frequencies of CD45RA+ CD45RO+ Tregs. Data represent mean ± SD from pooled samples of 3 independent experiments with 3 different PBMC donors. Statistical analysis was performed using One-way ANOVA with a Tukey’s multiple comparisons test reference to the control CD25- PBMC alone (****p<0.0001, *** p<0.001, and **p<0.01). (F) Quantification of Treg induction by MAPC cells at 2:1 and 4:1 PBMC: MAPC cell ratios cultured in direct contact (black circles) or in Transwell (gray squares). Data represented as mean ± SD from polled samples of 3 independent experiments with 3 different PBMC donors. Statistical analysis was performed using Two-way ANOVA with Tukey’s multiple comparisons test.

## Slide 4
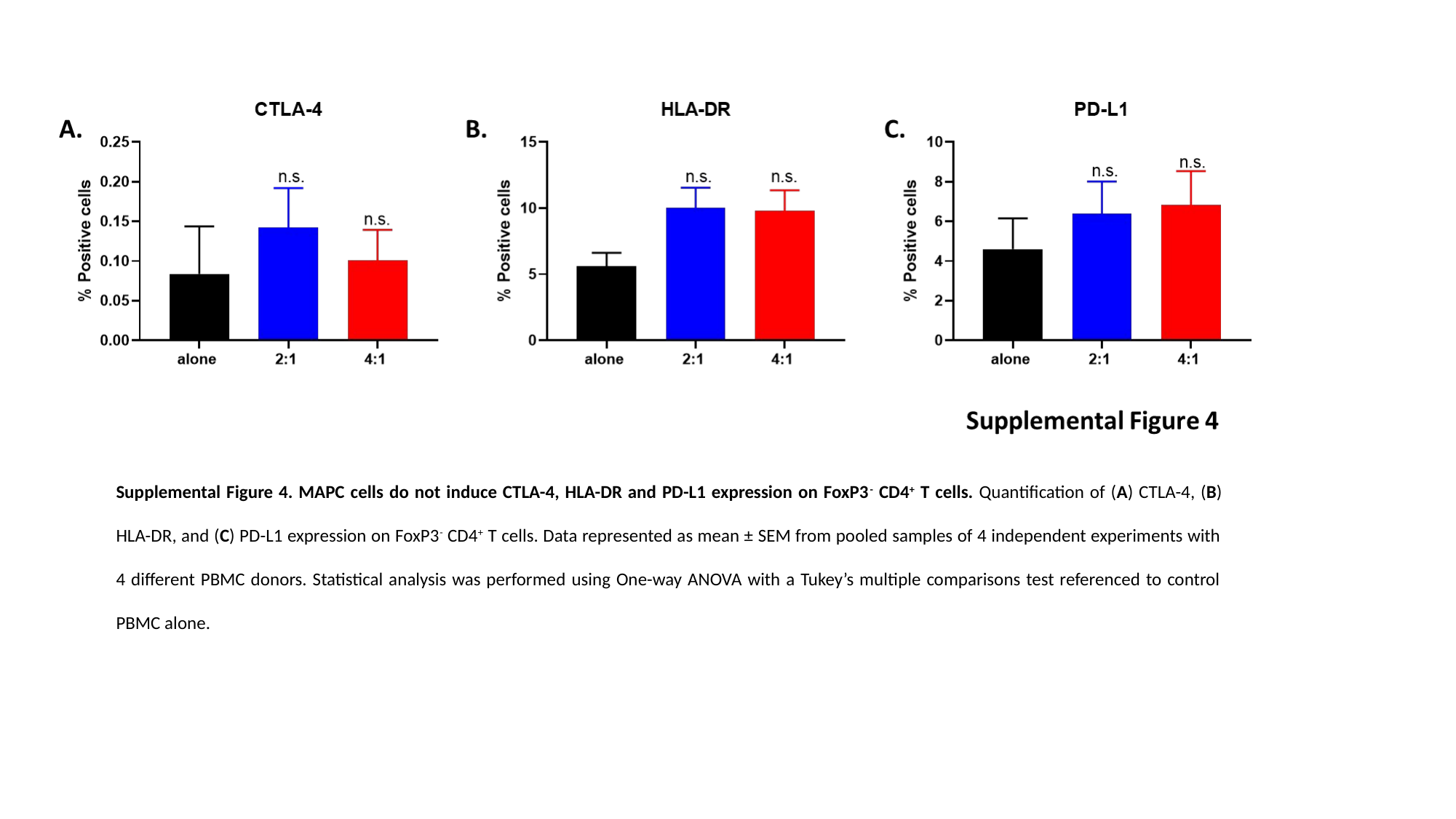

Supplemental Figure 4. MAPC cells do not induce CTLA-4, HLA-DR and PD-L1 expression on FoxP3- CD4+ T cells. Quantification of (A) CTLA-4, (B) HLA-DR, and (C) PD-L1 expression on FoxP3- CD4+ T cells. Data represented as mean ± SEM from pooled samples of 4 independent experiments with 4 different PBMC donors. Statistical analysis was performed using One-way ANOVA with a Tukey’s multiple comparisons test referenced to control PBMC alone.

## Slide 5
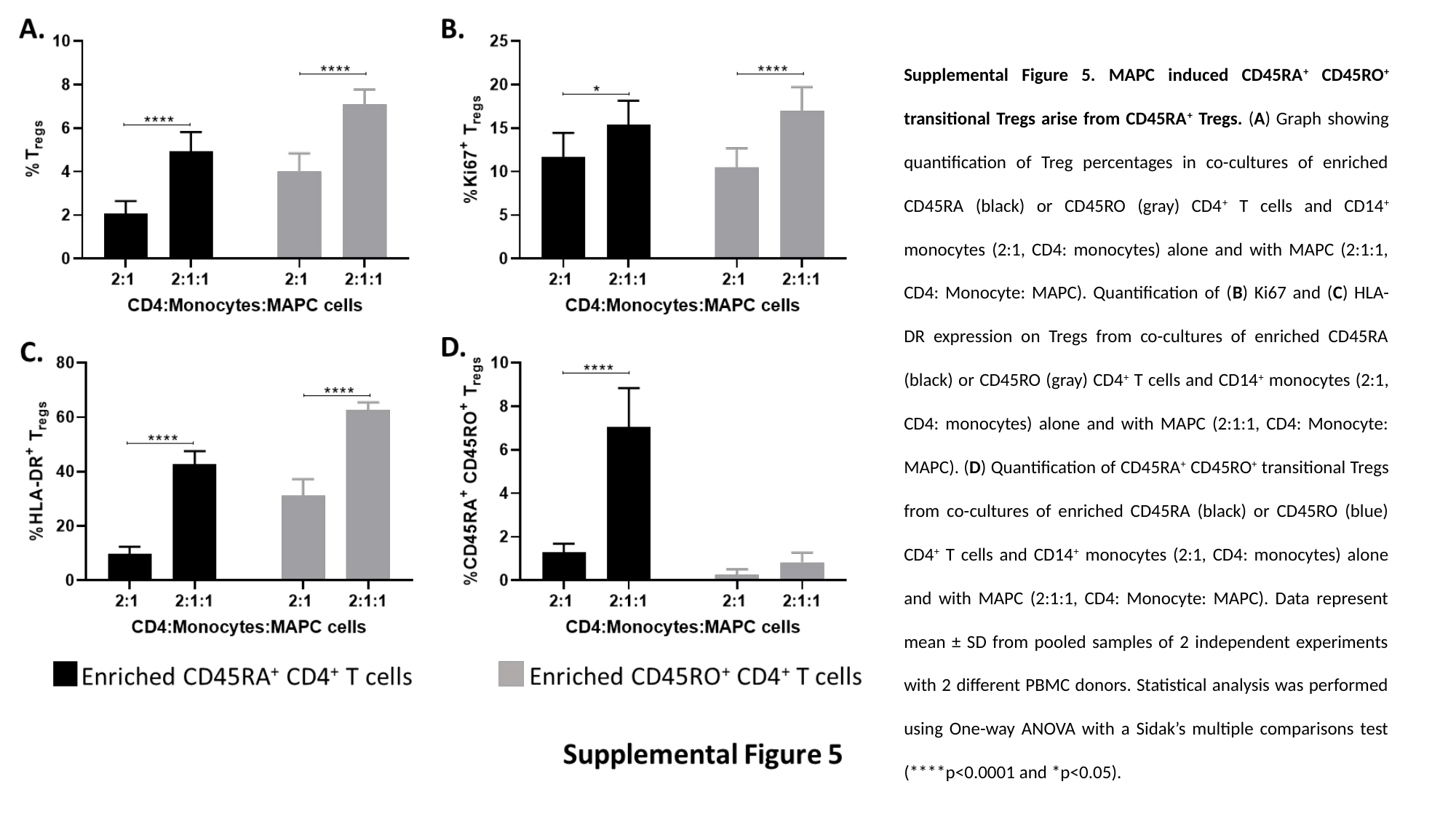

Supplemental Figure 5. MAPC induced CD45RA+ CD45RO+ transitional Tregs arise from CD45RA+ Tregs. (A) Graph showing quantification of Treg percentages in co-cultures of enriched CD45RA (black) or CD45RO (gray) CD4+ T cells and CD14+ monocytes (2:1, CD4: monocytes) alone and with MAPC (2:1:1, CD4: Monocyte: MAPC). Quantification of (B) Ki67 and (C) HLA-DR expression on Tregs from co-cultures of enriched CD45RA (black) or CD45RO (gray) CD4+ T cells and CD14+ monocytes (2:1, CD4: monocytes) alone and with MAPC (2:1:1, CD4: Monocyte: MAPC). (D) Quantification of CD45RA+ CD45RO+ transitional Tregs from co-cultures of enriched CD45RA (black) or CD45RO (blue) CD4+ T cells and CD14+ monocytes (2:1, CD4: monocytes) alone and with MAPC (2:1:1, CD4: Monocyte: MAPC). Data represent mean ± SD from pooled samples of 2 independent experiments with 2 different PBMC donors. Statistical analysis was performed using One-way ANOVA with a Sidak’s multiple comparisons test (****p<0.0001 and *p<0.05).

## Slide 6
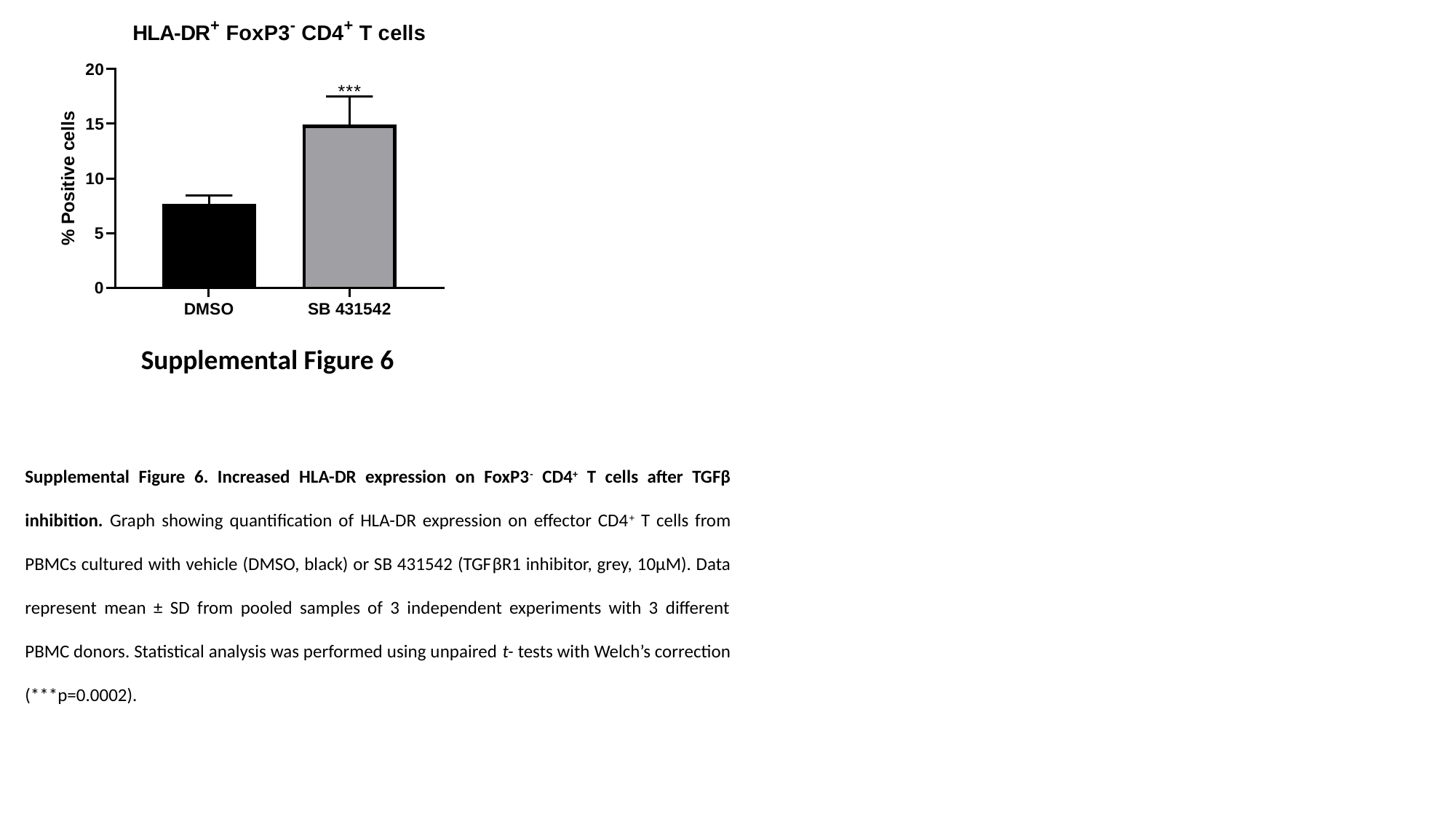

Supplemental Figure 6
Supplemental Figure 6. Increased HLA-DR expression on FoxP3- CD4+ T cells after TGFβ inhibition. Graph showing quantification of HLA-DR expression on effector CD4+ T cells from PBMCs cultured with vehicle (DMSO, black) or SB 431542 (TGFβR1 inhibitor, grey, 10µΜ). Data represent mean ± SD from pooled samples of 3 independent experiments with 3 different PBMC donors. Statistical analysis was performed using unpaired t- tests with Welch’s correction (***p=0.0002).

## Slide 7
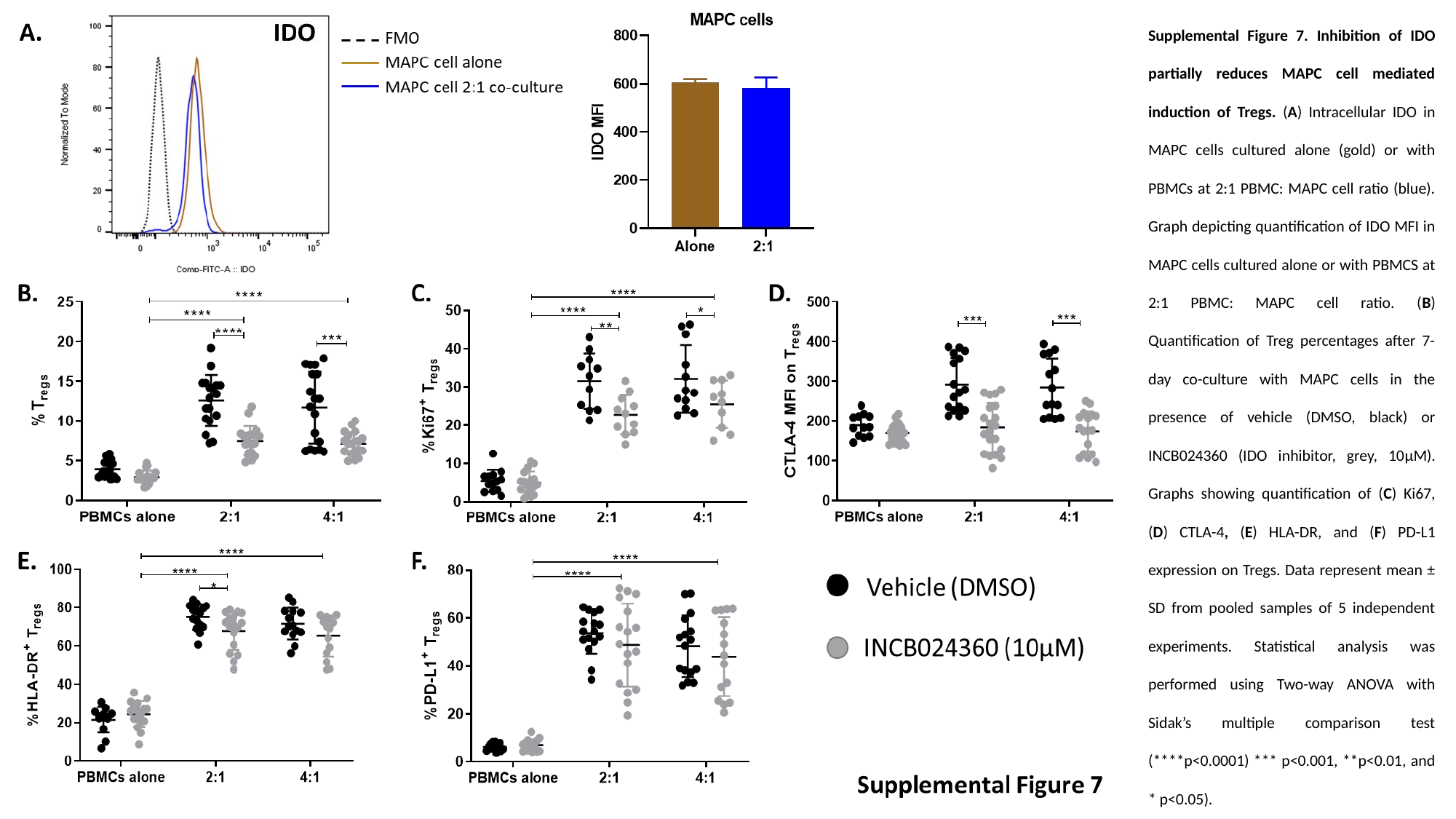

Supplemental Figure 7. Inhibition of IDO partially reduces MAPC cell mediated induction of Tregs. (A) Intracellular IDO in MAPC cells cultured alone (gold) or with PBMCs at 2:1 PBMC: MAPC cell ratio (blue). Graph depicting quantification of IDO MFI in MAPC cells cultured alone or with PBMCS at 2:1 PBMC: MAPC cell ratio. (B) Quantification of Treg percentages after 7-day co-culture with MAPC cells in the presence of vehicle (DMSO, black) or INCB024360 (IDO inhibitor, grey, 10μM). Graphs showing quantification of (C) Ki67, (D) CTLA-4, (E) HLA-DR, and (F) PD-L1 expression on Tregs. Data represent mean ± SD from pooled samples of 5 independent experiments. Statistical analysis was performed using Two-way ANOVA with Sidak’s multiple comparison test (****p<0.0001) *** p<0.001, **p<0.01, and * p<0.05).

## Slide 8
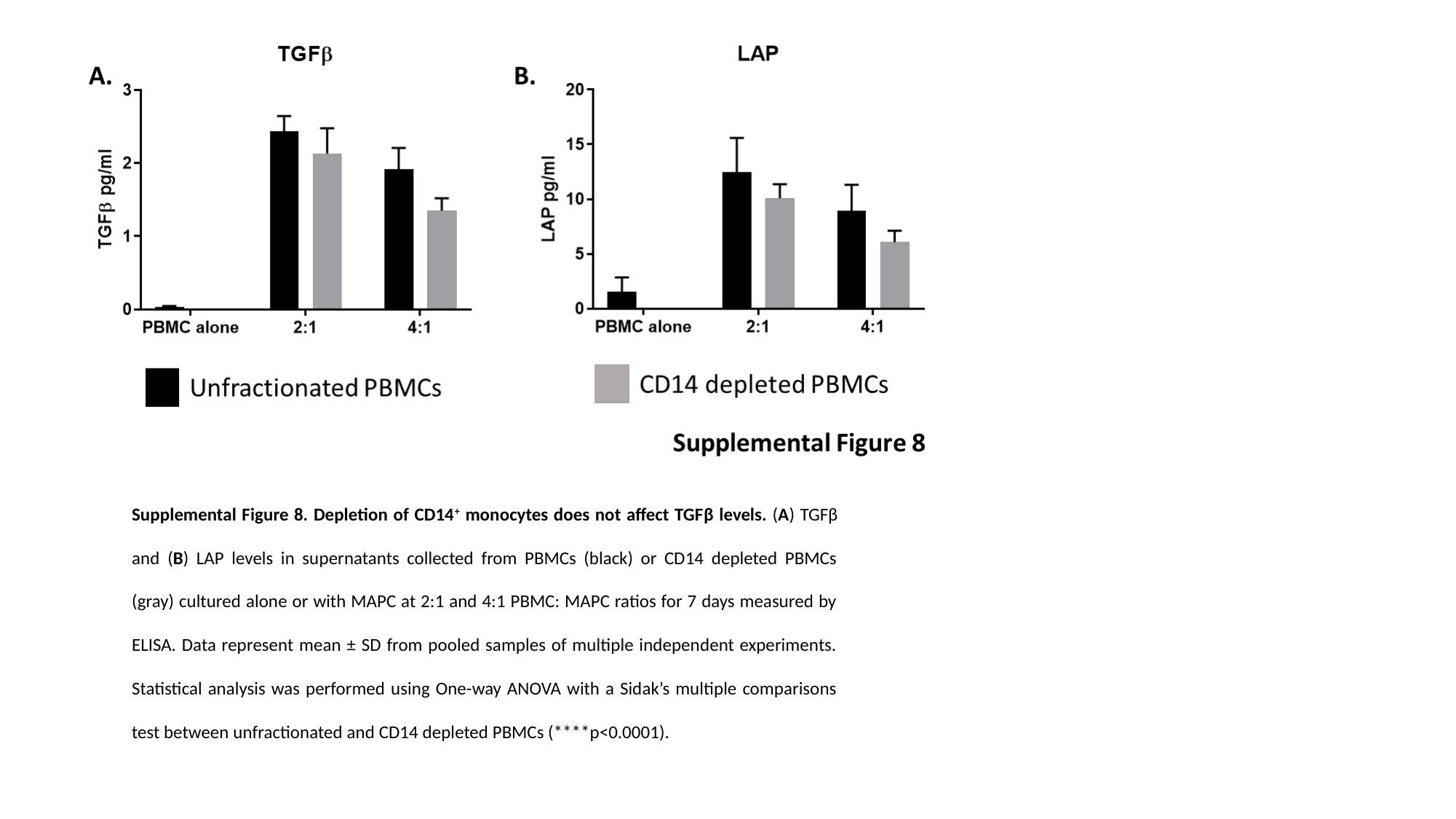

Supplemental Figure 8. Depletion of CD14+ monocytes does not affect TGFβ levels. (A) TGFβ and (B) LAP levels in supernatants collected from PBMCs (black) or CD14 depleted PBMCs (gray) cultured alone or with MAPC at 2:1 and 4:1 PBMC: MAPC ratios for 7 days measured by ELISA. Data represent mean ± SD from pooled samples of multiple independent experiments. Statistical analysis was performed using One-way ANOVA with a Sidak’s multiple comparisons test between unfractionated and CD14 depleted PBMCs (****p<0.0001).

## Slide 9
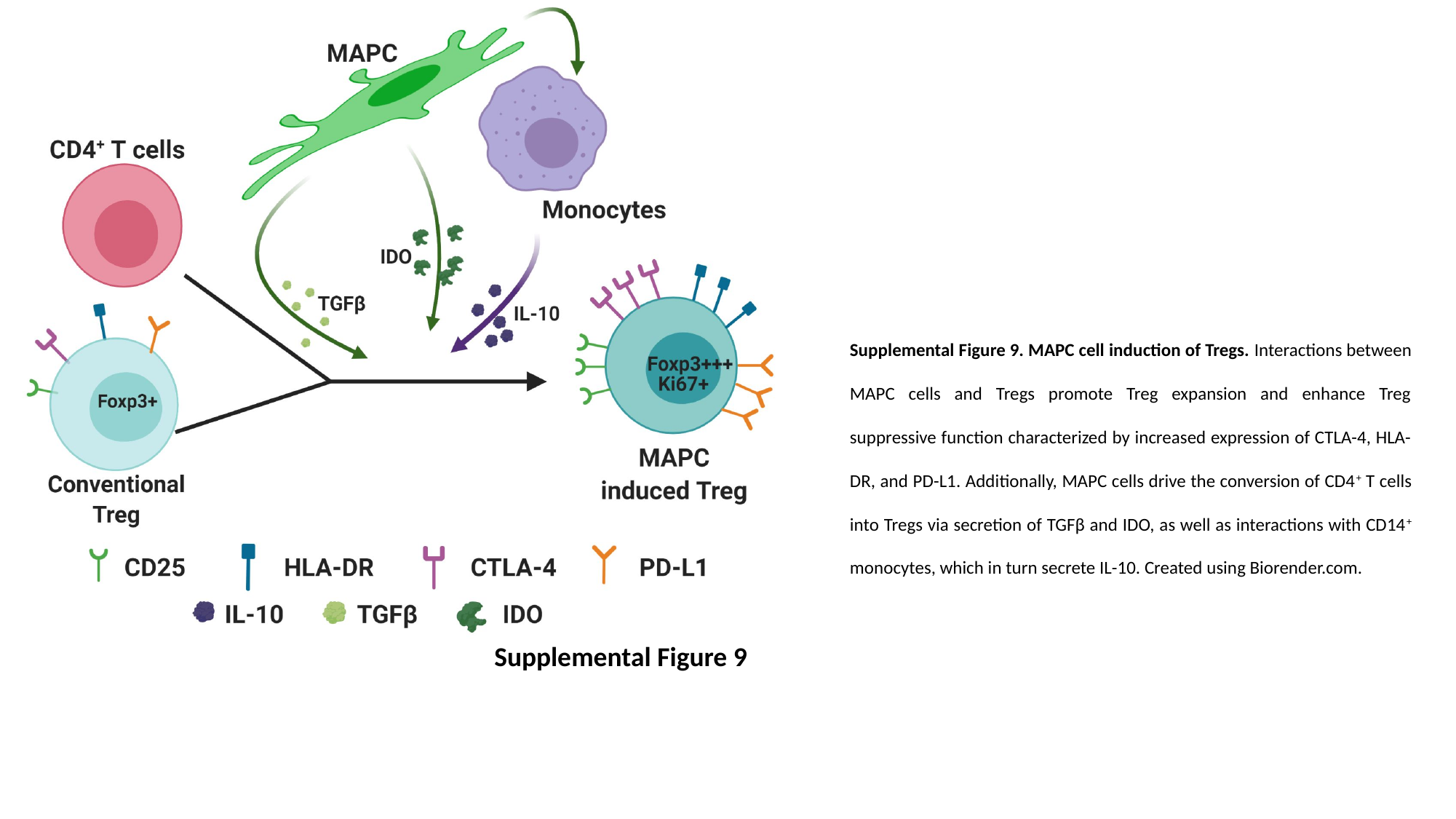

Supplemental Figure 9
Supplemental Figure 9. MAPC cell induction of Tregs. Interactions between MAPC cells and Tregs promote Treg expansion and enhance Treg suppressive function characterized by increased expression of CTLA-4, HLA-DR, and PD-L1. Additionally, MAPC cells drive the conversion of CD4+ T cells into Tregs via secretion of TGFβ and IDO, as well as interactions with CD14+ monocytes, which in turn secrete IL-10. Created using Biorender.com.
